# Recent Advancements in the Field of Chitosan/Cellulose-Based Nanocomposites for Maximizing Arsenic Removal from Aqueous Environment

**DOI:** 10.1021/acsomega.3c09713

**Published:** 2024-06-17

**Authors:** Kalpana Chauhan, Prem Singh, Kshipra Sen, Rakesh Kumar Singhal, Vijay Kumar Thakur

**Affiliations:** † Chemistry under School of Engineering and Technology, Central University of Haryana, Mahendragarh, Haryana 123031, India; ‡ Shoolini University, Solan, Himachal Pradesh 173229, India; § Analytical Chemistry Division, Bhabha Atomic Research Centre, Mumbai 400085, India; ∥ Biorefining and Advanced Materials Research Centre, Scotland’s Rural College (SRUC), Kings Buildings, West Mains Road, Edinburgh EH9 3JG, United Kingdom

## Abstract

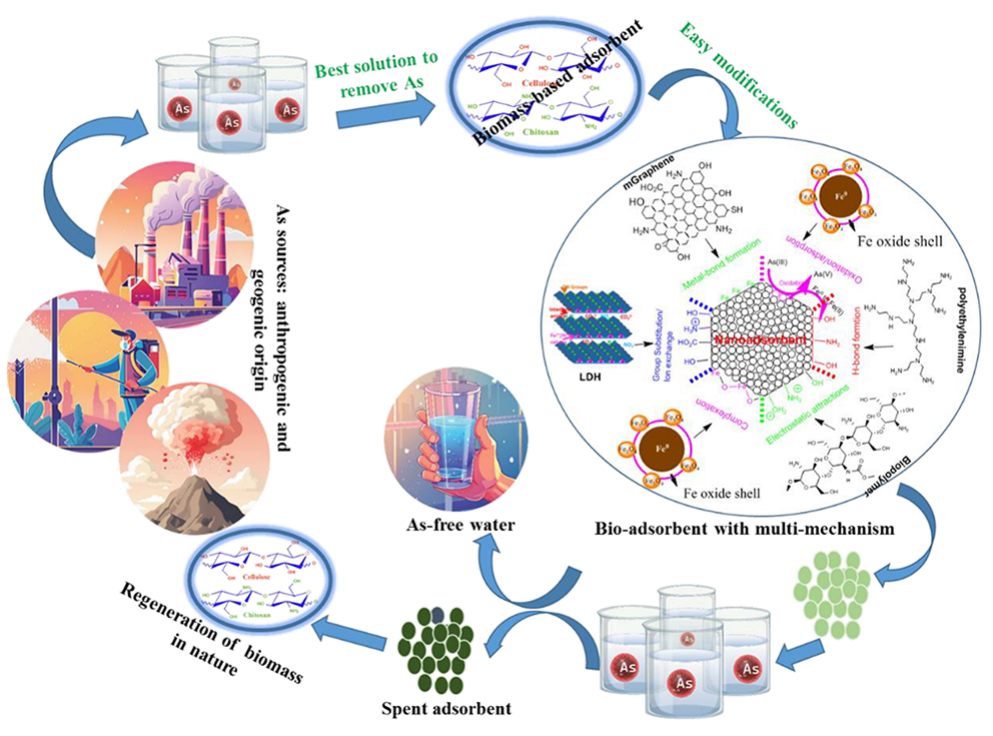

Water remediation, acknowledged as a significant scientific topic, guarantees the safety of drinking water, considering the diverse range of pollutants that can contaminate it. Among these pollutants, arsenic stands out as a particularly severe threat to human health, significantly compromising the overall quality of life. Despite widespread awareness of the harmful effects of arsenic poisoning, there remains a scarcity of literature on the utilization of biobased polymers as sustainable alternatives for comprehensive arsenic removal in practical concern. Cellulose and chitosan, two of the most prevalent biopolymers in nature, provide a wide range of potential benefits in cutting-edge industries, including water remediation. Nanocomposites derived from cellulose and chitosan offer numerous advantages over their larger equivalents, including high chelating properties, cost-effective production, strength, integrity during usage, and the potential to close the recycling loop. Within the sphere of arsenic remediation, this Review outlines the selection criteria for novel cellulose/chitosan-nanocomposites, such as scalability in synthesis, complete arsenic removal, and recyclability for technical significance. Especially, it aims to give an overview of the historical development of research in cellulose and chitosan, techniques for enhancing their performance, the current state of the art of the field, and the mechanisms underlying the adsorption of arsenic using cellulose/chitosan nanocomposites. Additionally, it extensively discusses the impact of shape and size on adsorbent efficiency, highlighting the crucial role of physical characteristics in optimizing performance for practical applications. Furthermore, this Review addresses regeneration, reuse, and future prospects for chitosan/cellulose-nanocomposites, which bear practical relevance. Therefore, this Review underscores the significant research gap and offers insights into refining the structural features of adsorbents to improve total inorganic arsenic removal, thereby facilitating the transition of green-material-based technology into operational use.

## 1 Introduction: Arsenic Contamination - A Significant Environmental Challenge

The inadequacy of clean water has become a global issue. Currently, around the world, more than 1.2 billion people lack access to clean drinking water.^1^ The rapid growth in population, urbanization, and climate disruption has contributed to the overexploitation of water resources, as well as an alarming rise of toxic pollutants in aqueous systems due to depleting water tables.^2,3^ Eventually, this has increased the demand for clean water.^4^ The most common water pollutants are inorganic, organic, and biological (like pharmaceuticals, oils, phenols, pesticides, detergents, fertilizers, greases, microbial pathogens, heavy metals, microplastics, etc.).^5−7^ However, one of the most pressing challenges in water purification arises from the removal of heavy metal ions, particularly As, Hg, Cd, Pb, Cr, among others.^8,9^ These heavy metals pose a significant threat as they permeate into the human food chain, owing to their high solubility in water and subsequent accumulation in the environment due to their persistent nature.^10,11^ Tackling the issue of heavy metal presence poses a daunting challenge for both researchers and industries, as natural biological processes lack the capability to break them down.^10,12^ Arsenic stands out as the most toxic among these heavy metals, causing a significant global environmental concern.^13,14^ Reports indicate that over 200 million people across 50 countries regularly consume arsenic-contaminated water, which is above the WHO recommended permissible limit of 10 parts per billion (ppb).^15,16^

Arsenic, a metalloid, is present in both organic and inorganic species in the environment.^17^ Inorganic species tend to have higher concentrations and toxicity compared to organic ones.^18^ In an aqueous environment, inorganic species can exist in various oxidation states (−3, 0, +3, and +5).^19,20^ Among these, arsenite [As(III)] is significantly more toxic than arsenate [As(V)], exhibiting 20–60 times higher toxicity due to its higher mobility in the environment and faster cellular uptake.^21^ Redox potential and pH play crucial roles in determining the distribution of As(III) and As(V) species as As(V) typically dominates in alkaline and oxidizing groundwater, forming oxyanions (H_2_AsO_4_^–^ and HAsO_4_^2–^) at pH levels below 6.9, whereas As(III) prevails in moderately reducing the aqueous environment as neutral H_3_AsO_3_ at pH levels below 9.2.^22−24^

Arsenic contamination in aqueous environment stems through a combination of natural processes, including rock weathering, geochemical reactions, biological activities, volcanic emissions, and anthropogenic activities, such as industrial mining, metallurgical industries, and the burning of fossil fuels, as well as the use of arsenic-based pesticides and herbicides.^25,26^ Naturally occurring arsenic-bearing minerals, particularly arsenopyrite (FeAsS), serve as the foremost source of arsenic contamination in drinking water and natural water bodies.^27,28^ These minerals are commonly found in anaerobic conditions and various rock-forming minerals, including sulfide, oxide, phosphate, carbonate, and silicate with varying concentrations.^29,30^ Among these minerals, iron and aluminum oxides in sediments play a pivotal role in arsenic contamination of groundwater. This is primarily due to the process of reductive dissolution of these oxides, which serves as the main mechanism for the release and mobility of arsenic in groundwater.^31,32^

Arsenic accumulation, even in trace levels, can have severe adverse effects on human health.^33^ Groundwater, a primary source of drinking water, is considered the main route of exposure to inorganic arsenic. Food is the second-largest contributor to arsenic ingestion after direct consumption of contaminated water.^34,35^ In fact, approximately 25% of arsenic in food is inorganic. Long-term exposure or a high intake of arsenic-contaminated water can lead to various health issues in humans, including cardiovascular diseases, skin lesions, lung cancer, kidney cancer, bladder cancer, neurological disorder, muscular weakness, and even death.^36−38^ Arsenic toxicity affects approximately 200 enzymes, particularly those involved in the cellular corridor like DNA replication and repair, ultimately leading to a reduction in ATP formation. Moreover, unmetabolized arsenic species produced during redox cycling and metabolic activities can generate reactive oxygen intermediates, which may consequently harm lipids and DNA.^39^

### 1.1 Traditional Methods: Adsorption Emerges as a Suitable Approach

Numerous methods including physical, chemical, and biological approaches have demonstrated effectiveness in removing arsenic from aqueous solutions at the laboratory scale, including precipitation/coprecipitation, coagulation/flocculation, membrane filtration, oxidation, adsorption, and ion-exchange.^40−42^ Nonetheless, each of these technologies comes with its unique advantages along with challenges or concerns, as is given in Table 1. Most of these techniques involve high costs, complex processes, ongoing maintenance, and the generation of significant volumes of toxic sludge, rendering them unsuitable for small communities with limited resources.^43−45^ Moreover, most of these practices are efficient in removing As(V) but may require a pretreatment step, such as peroxidation, to oxidize As(III) to As(V) for effective arsenic removal.^46^ Also, the aforementioned techniques have gradually lost appeal due to their effectiveness primarily at higher concentrations of contamination and their inability to meet revised drinking-water standards for trace contamination levels.

**Table 1:** Merits and Demerits of Different Treatment Methods in Arsenic Removal^47^

methods	positive	negative
ion exchange	high specificity, low sensitivity to water pH	technique requires expensive media, such as strong base anion exchange resins, high-tech maintenance or operation, and primarily useful at low-total dissolved solids (TDS) levels; removal of As(III) presents a challenge, and disposal of sludge is also an issue
adsorption	widely employed technique in commerce, less expensive, achieve almost 95% removal efficiency, offers simple handling and operation, and a sludge-free process	critically depends on the solution’s pH, necessitates regular replenishment of adsorbent materials, and creates relatively low solid hazardous waste
chemical precipitation	process involves common chemicals, simple operation, and relatively minimal capital costs	principally eliminates As(V) and generates poisonous sludge; pretreatments such as preoxidation, prechlorination are necessary to enhance removal efficiency, potentially leading to the formation of byproducts and odor contamination
membrane technique	approach requires no chemicals, generates no hazardous solid wastes, capable of eliminating bacteria and other pollutants	high maintenance and operation cost, require pretreatment, risk of producing hazardous wastewater
electrocoagulation	approach requires no chemicals, efficient and simple to maintain	requires a strong foundation to be commercially viable, necessitates significant power consumption on a large scale
phytoremediation	an ecologically sound approach that is free of chemicals	requires a strong foundation to become commercially viable

Among the aforementioned technologies, the adsorption technique has gained significant attention owing to its simplicity in operation, cost-effectiveness, high efficiency, and sludge-free operation. Its appeal is further augmented by the availability of diverse adsorbents ranging from organic, inorganic to biosorbents, rendering it a versatile option suitable for both small-scale and large-scale applications.^48−50^

Adsorption, fundamentally a surface phenomenon, entails the interaction of contaminants or adsorbates within the surrounding medium with the surface of adsorbent through various mechanisms, such as π–π interactions, physical bonds (e.g., van der Waals forces, hydrogen bonding, electrostatic attractions), as well as chemical bonds (e.g., ion exchange and complexation). Understanding these mechanisms is crucial as they dictate the efficacy and specificity of the adsorption process. At lower temperatures, physisorption dominates, enabling the formation of multilayer adsorption. Physisorption is characterized by weaker interactions between the adsorbate and adsorbent, primarily governed by physical forces. On the other hand, chemisorption, also recognized as activated adsorption, occurs predominantly at higher temperatures, typically forming monolayer sorption. Chemisorption involves stronger chemical bonds between the adsorbate and the adsorbent, resulting in higher selectivity and specificity. However, the challenge of effectively removing trace levels of geogenic contaminates remains a significant concern today, necessitating the development of specialized low-cost adsorption media to address this issue.^51^

**Figure 1:**
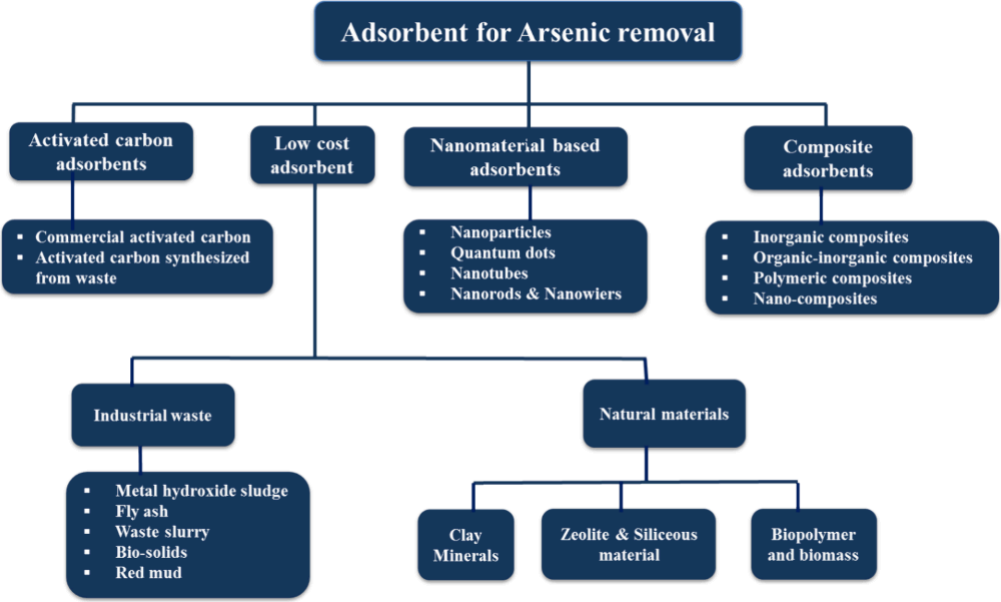
Different types of possible adsorbents for arsenic removal.

In adsorption technology, the choice of an appropriate adsorbent is crucial. An effective adsorbent should possess specific properties such as a large surface area, rapid adsorption rate, and fast equilibrium time.^52^ However, the complexity of arsenic adsorption arises due to its varied speciation in different aqueous conditions. For example, iron oxides exhibit a preference for adsorbing As(V) under pH 5–6 compared to As(III), while above pH 7–8, As(III) is adsorbed more favorably.^53^ Consequently, adsorption has proven as a viable technique for the remediation of both As(III) and As(V), aligning with WHO standards. Besides, adsorption is straightforward to implement and does not necessitate the addition of chemicals, making it a practical and cost-effective approach for treating arsenic-contaminated water comprehensively. Additionally, for sustainable technology implementation, characteristics such as minimal processing energy and sustainable materials resourcing are also critical. Figure 1 illustrates the different types of adsorbents, which can be used in arsenic removal applications.

#### 1.1.1 Nature-Based Adsorbents: Superior Alternatives for Commercially Viable Adsorbents

In recent times, amidst growing environmental concerns and the pressing need for sustainable water treatment options, natural materials have emerged as a prominent area of focus for researchers to removal heavy metal ions from aqueous environments. Especially, biopolymeric adsorbents possess tailored structures, adjustable surface groups, regeneration capabilities, and biodegradable properties, rendering them promising candidates for heavy metal adsorption with minimal environment impact.^54^ However, despite their unique characteristics, biopolymeric adsorbents encounter certain challenges, such as low adsorption capacity, lack of selectivity, poor mechanical strength, and a tendency to swell significantly in aqueous systems, which limit their practical relevance.^55^ Among biopolymers, including alginate, cellulose, chitin, and pectin, several have demonstrated promising efficiency for arsenic removal in laboratory settings.^11,56,57^ Alginate, with its carboxyl groups, and pectin, with its functional groups (−COOH/R), can form complexes with arsenic ions, making them potential adsorbents.^58^ However, mechanical strength might be an issue for commercial aspects.^59^

Like biopolymers, natural metal oxides, such as iron oxides [iron-based nanoparticles, iron-based layered double hydroxides (LDHs), zerovalent iron (ZVI), oxy-hydroxides, and others], clays, and zeolites, also offer attractive adsorption potential for arsenic removal. They are cost-effective, exhibit high adsorption efficiencies, and are environmentally friendly.^60^ Importantly, the adsorption of arsenic by iron oxide and alumina is a naturally occurring process. Moreover, iron-based media, such as granular ferric oxide (GFO) and granular ferric hydroxide (GFH), have been commercially utilized for small drinking water systems.^61,62^

Incorporating size control in metal oxides, particularly zerovalent iron particles, further enhances their performance in arsenic removal. This improvement can be attributed to several distinctive advantages, including high specific surface area, reactivity, specificity, and shortened intraparticle diffusion pathways.^63−67^ On small size scales, adsorbent materials exhibit numerous exposed sites due to their large specific surface area, resulting in significantly shorter removal times compared to their bulk form counterparts.^68,69^ However, despite the proven efficiency of natural adsorbents in arsenic adsorption, practical field applications are often limited due to the presence of interfering ions or complex water systems. Nonetheless, the strong chemical affinity between the adsorbent and arsenic can mitigate the complexity of the environment, thereby providing a specialized adsorbent with dynamic applicability.^70^

#### 1.1.2 Chitosan/Cellulose: Preferred Components in Adsorbents for Commercial Water Treatment

**Figure 2:**
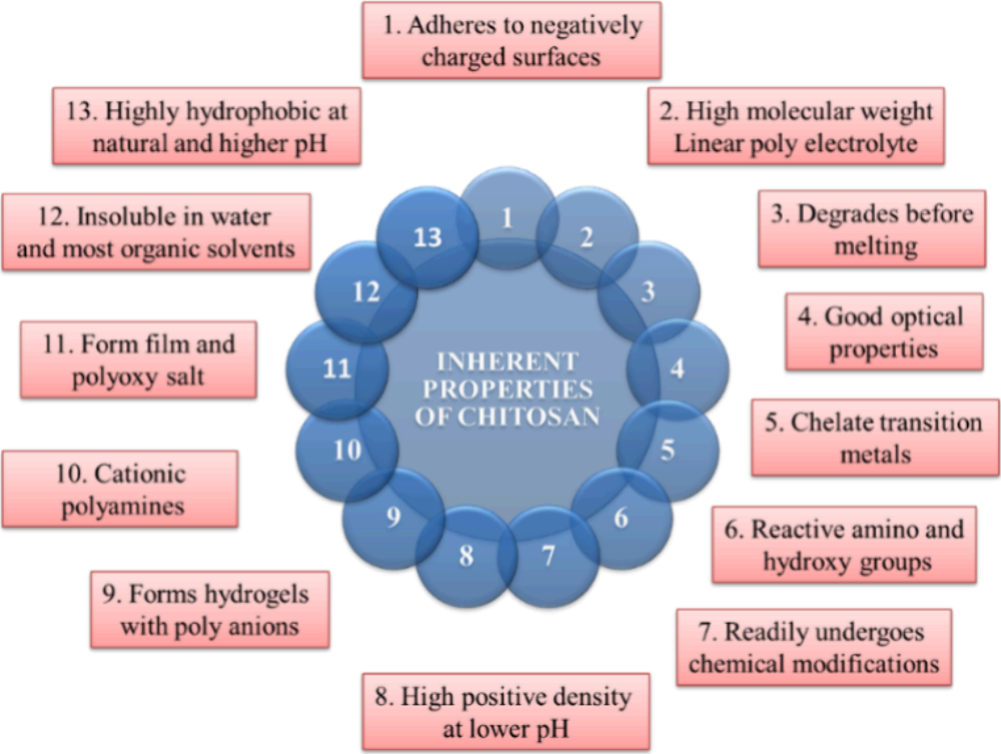
Chitosan inherent properties.

Commercially available chitosan, derived from chitin, the second most abundant natural biopolymer after cellulose, typically possesses a deacetylation level of 75%. It stands out among bioadsorbents due to several critical inherent properties, such as superior chelating ability (attributed to −OH and −NH_2_ groups via coordination bond or ion exchange) and a flexible polymeric chain.^71−78^ Chitosan is comprised of d-glucosamine and *N*-acetyl-d-glucosamine structural units linked by β-(1–4)-linkages. However, despite its remarkable properties, the pristine structure of chitosan exhibits relatively low adsorption capacity and slow adsorption kinetics due to its low surface area, low porosity, and semicrystalline nature.^79−81^ Additionally, it presents challenges for practical applications across a broader pH range due to its solubility only in acidic medium.^82−88^ This aspect currently hinders its widespread success in achieving practical relevance. These attributes of chitosan are highlighted in Figure 2.

Physical and chemical modifications are effective strategies for augmenting the interaction sites, surface area, and pore volume of native chitosan. These enhancements enhance the accessibility of its functional groups, resulting in improved adsorption outcomes.^89−91^ Moreover, chitosan naturally proves superior chelation capabilities for cations due to the presence of amine groups, compared to other conventional carbohydrate-based biosorbents.^92−95^ On the other hand, the protonation of the amine groups in chitosan also facilitates anions sorption through ion-exchange or electrostatic attractions.^96−98^ As a result, the interaction properties of chitosan’s amine groups are influenced by various parameters, including the degree of deacetylation, neutralization, and pH of the solution, all contributing to improved adsorption efficiency.

Similarly, cellulose stands out as nature’s most abundant and renewable polymer with a myriad of advantageous properties, such as biodegradability, biocompatibility, and cost-effectivness.^99,100^ Its molecular structure is comprised of repeating β-d-glucopyranose units, featuring both amorphous and crystalline domains.^101^ One of cellulose’s distinguishing characteristic is the presence of three hydroxyl groups (primary/secondary-alcohol) in each glucopyranose unit, offering facile modification opportunities tailored to achieve desired applications. The reactivity of these hydroxyl groups varies across positions within the anhydroglucose unit of cellulose. Furthermore, cellulose exhibits remarkable physical and chemical properties, including high mechanical strength, stability, and flexibility, making it highly versatile for various materials and products.^102^ Recent studies indicate that cellulose-based adsorbents demonstrate adsorption capacities and mechanical strength comparable to those of ion exchange resins or commercially available activated carbon.^103^ Nonetheless, cellulose acetate, a derivative, has long been utilized in filtration media and has gained commercial prominence.^104^ Its nanofibrous membrane, in particular, presents a highly continuous, smooth, and interconnected porous structure, which is ideal for effectively filtering microorganisms in water treatment applications.^105^

**Figure 3:**
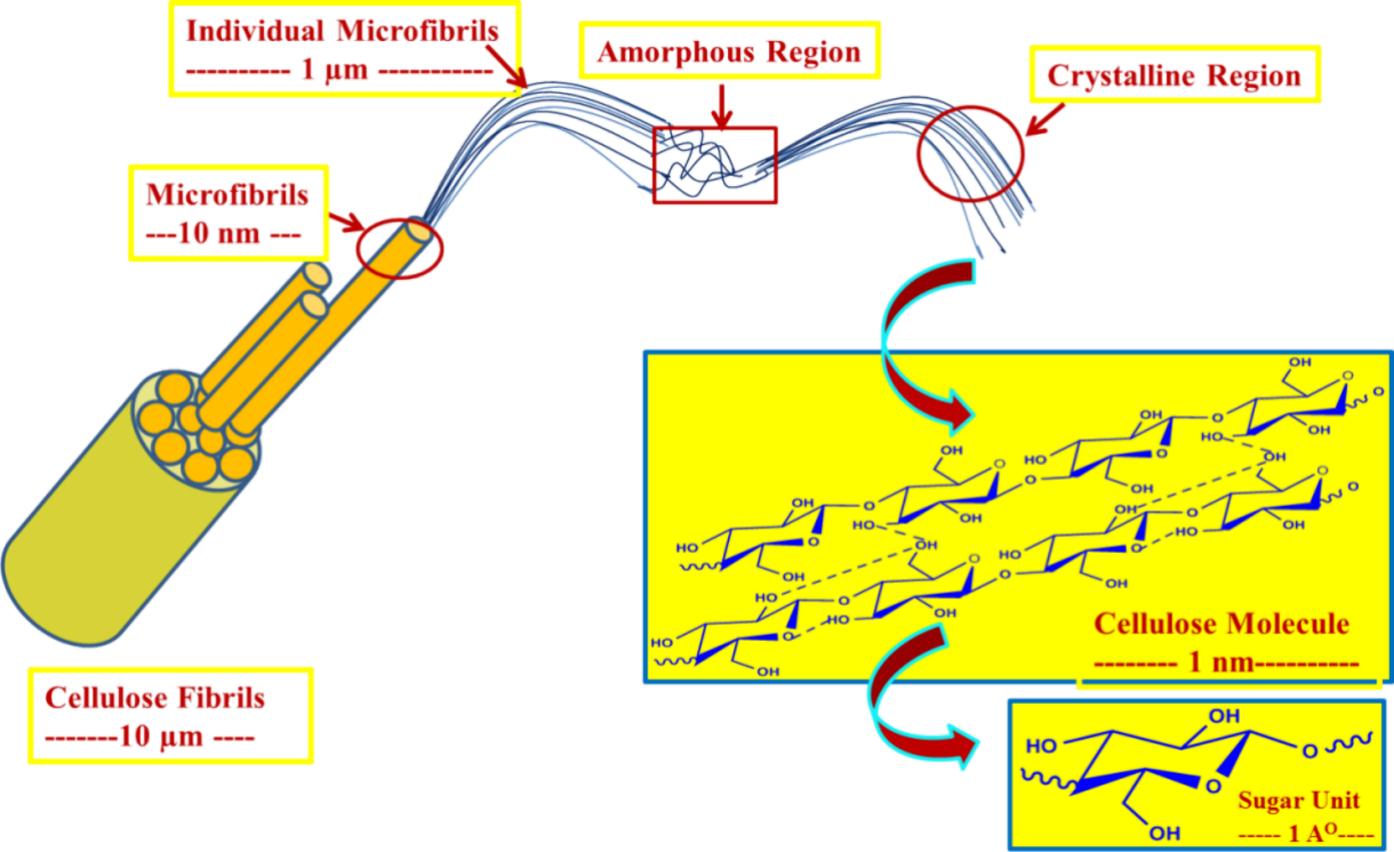
Cellulose unit structure and its nanomaterials in different morphologies. Reproduced or adapted with permission from ref (112). Copyright 2014 Elsevier.

**Figure 4:**
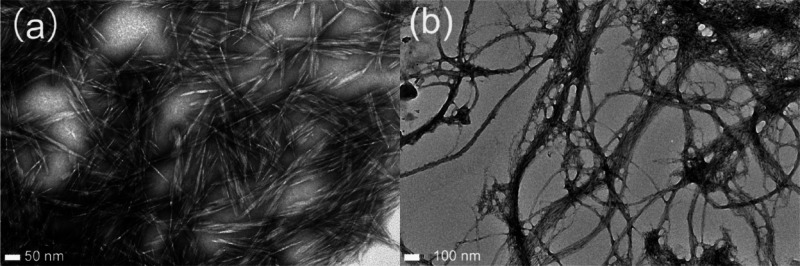
TEM images of (a) CNCs and (b) CNFs. Reproduced with permission from ref (113). Copyright 2013 American Chemical Society.

Over the past two decades, nanocellulosic structures have attained tremendous attention from both academia and industry, which can be scaled for industrial production.^106^ Nanocellulose can be broadly categorized into two types, irrespective of their production method: cellulose nanocrystals (CNCs) and cellulose nanofibrils (CNFs).^107−110^ These nanostructures can be derived from a variety of cellulosic sources, including wood, plants, tunicate, algae, and bacteria. CNCs are typically synthesized through acid hydrolysis, wherein the amorphous regions of cellulose are selectively broken down, leaving behind rod-like crystalline structures (Figure 4a). On the other hand, CNFs are produced through mechanical disintegration of cellulose pulp fibers (Figure 4b).^111^ A graphical presentation in Figure 3 depicts the transition from cellulose fibrils to the cellulose molecular level. Aforementioned nanocellulose structures possess a myriad of unique characteristics, including distinct morphology and geometrical dimensions, enhanced crystallinity, reinforcing capabilities, high-specific surface area, exceptional mechanical strength, stability, flexibility, improved surface chemical reactivity, and biocompatibility. Consequently, the versatility and exceptional properties of nanocellulose have opened up new avenues for innovation across a wide range of industries, including advanced materials and composites for different applications like water treatment, biomedical applications, and others.

In conclusion, cellulose and chitosan, being among the most abundant organic raw materials, have attracted significant interest in both academic research and industrial applications. This interest stems primarily from their inherent stability, sustainability, and mechanical strength. Notably, cellulose stands out as one of the most economically viable raw materials for developing environmentally friendly adsorbents. Moreover, one of the most facilitating aspects of cellulose/chitosan applications is their ability to form hydrogen bonds, enabling their potential regeneration into various physical forms such as beads, granules, fibers, gels, membranes, or films. This versatility enhances their practical relevance for large-scale commercialization.

##### 1.1.2.1 Enhancing Adsorption Competence through Functionalized Chitosan/Cellulose

**Figure 5:**
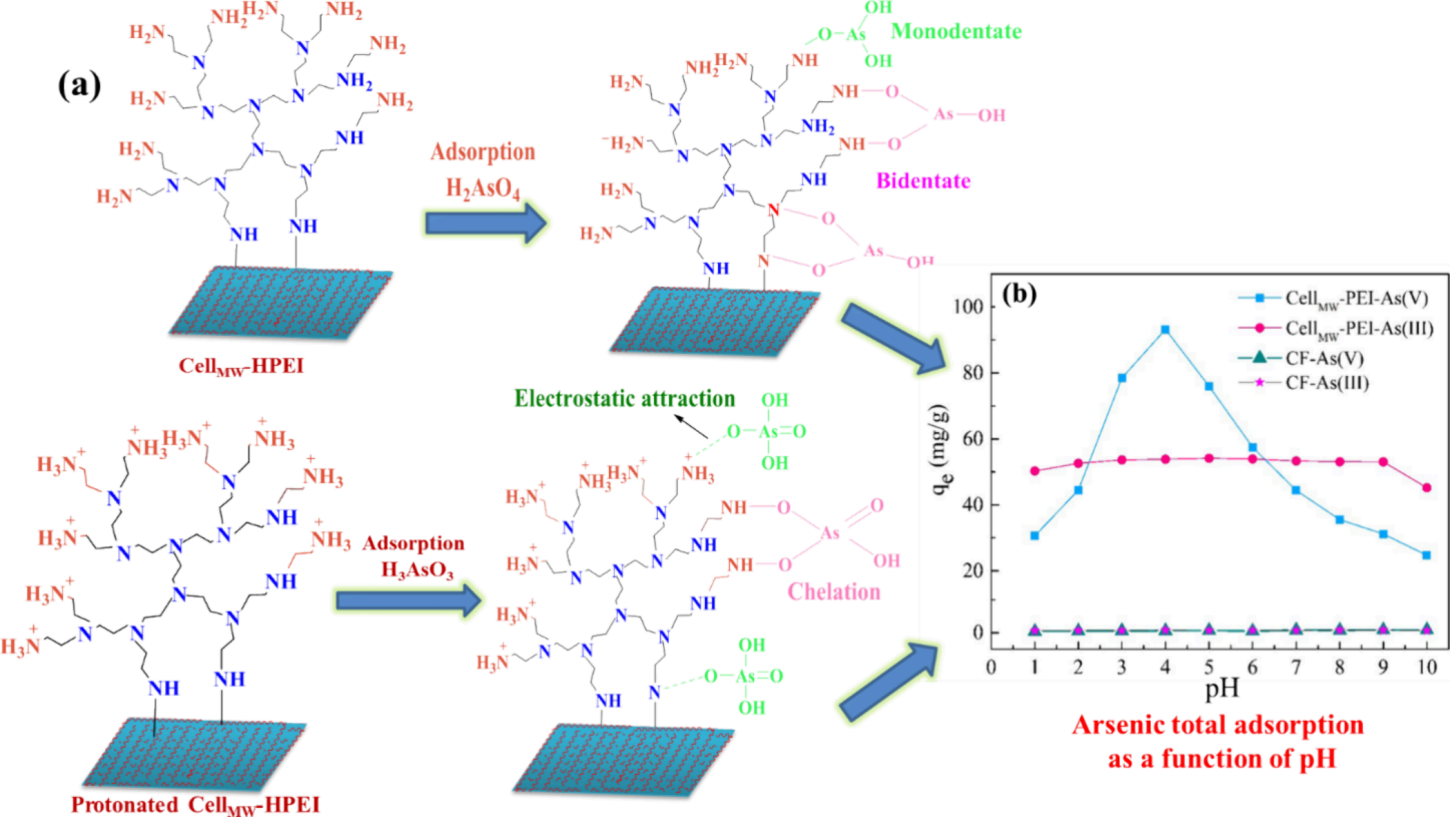
Possible interactions between (a) As(III/V) and cell_MW_-HPEI fibers and (b) arsenic adsorption results with pH. Reproduced or adapted with permission from ref (24). Copyright 2012 Royal Society of Chemistry.

The pristine chitosan has limited the sorption prospective for arsenic, particularly for As(III) compared to As(V). However, the chemical modification of chitosan by integrating high-affinity functionalities (−NH_2_, −SH, −OH, −CO_2_H, etc.) enhances its sorption capabilities. This improvement occurs through enhanced interactions between arsenic and high-affinity functionalities via mechanisms such as electron donation, cation exchange, Lewis acid–base interaction, and surface complexation resulting in more effective arsenic removal.^114,115^ Furthermore, the interaction between these functional groups and metal ions adheres to the principles of hard and soft acids and bases. In this context, hard acids exhibit a preference for coordinating with hard bases, while soft acids tend to coordinate with soft bases.^116^ Particularly, nitrogen group-based functionalization plays a crucial role in augmenting arsenic removal efficiency, wherein the −NH_2_ group enables electrostatic interactions at pH 4.0 for As(V), while monodentate and bidentate complexes aid in As(III) removal at neutral pH. For example, pyridinium-modified chitosan exhibits superior efficiency and sustainability compared to conventional coagulants like FeCl_3_ in arsenic removal, especially when employed in coagulation-like systems akin to conventional water treatment processes.^117^ Cellulose-polyethylenimine (cell_MW_-HPEI), incorporating hyperbranched PEI (polyethylenimine) within the cellulose structure in high −NH_2_ group density, also offers impressive adsorption capacities of 54.13 mg/g for As(III) and 99.35 mg/g for As(V), as illustrated in Figure 5.^24^

Controlling the size of the adsorbent is even more essential to enable achieving enhanced adsorption interaction sites on the adsorbent surface. For instance, nanofibers, with diameters of approximately 5 nm, offer a significantly larger theoretical surface area for functionalization per gram compared to microfibers, which typically have diameters around 30 μm. This difference results in a higher density of interaction sites on the nanofiber surface. A study investigates the impact of size on cysteine content in cysteine-functionalized micro- and nanofibers, where both micro- and nanofibers exhibit tethered −SH groups on their surfaces for arsenic removal. Despite observing aggregation in cysteine-functionalized nanofibers, comparable As(III) removal efficiencies were witnessed due to similar thiol (sulfur) content between micro- and nanofibers.^118^ Even the electrospun nanofiber membrane (ENM) of chitosan-functionalized-poly(vinyl alcohol)/sodium alginate (CS-f-PVA/SA) revealed that uniform nanofiber formation enhances the maximum Langmuir adsorption capacity for As(III) to 540.40 mg/g, even at a low concentration of 400 ppb.^119^ Thus, the fibrous adsorbent structures have gained prominence due to their promising characteristics, including minimal agglomeration, high surface area, improved porosity, and excellent binding capability for pollutants.

Furthermore, the attachment of specific functionalities with strong affinity to biopolymeric structures facilitates the simultaneous removal of both As(III) and As(V) from aqueous systems. Remarkably, nearly equal capacities of 17.0 and 17.6 mg g^–1^ at 50 ppb for As(III) and As(V), respectively, have been achieved without requiring any pretreatment for As(III).^120^ However, enhanced kinetics, stability, and adsorption potential, even at trace levels, are crucial practical aspects.

Better kinetics in adsorption applications can be achieved with nanomaterial, as adsorption rates are inversely proportional to the square root of particle radius, according to Frick’s law of diffusion.^121^ However, despite impressive adsorption performance characteristics, nanosized materials also pose several shortcomings, including difficulties in separation, complex syntheses requiring additional energy on a large-scale, challenges in regeneration/reusability, particle aggregation, lack of stability, and potential risks to human health and the ecosystem due to environmental release.^122−124^ Nonetheless, nanocomposites hold promise in addressing many of these challenges in the real world, offering excellent future prospects.^125−127^ Therefore, the subsequent part of this Review will summarize literature on bionanocomposites to elucidate the role of size and structure in arsenic adsorption. The development of low-cost nanocomposites based on biopolymer is an interesting area for researchers to explore economical solutions for commercial water treatment.^128−133^

Numerous comprehensive review articles have been published on the biosorption of arsenic from aqueous systems.^50,134,135^ Nevertheless, in this Review, our foremost motivation is on the structure and applications of chitosan/cellulose-based nanocomposites for removing arsenic from aqueous systems. Cellulose and chitosan’s abundance and ease of processing make them cost-effective compared to other biopolymers, essential for large-scale applications. This Review also explores advancements in biocomposites for comprehensive arsenic removal, summarizing relevant adsorption findings, discussing key factors like pH, reusability, and stability, and highlighting future prospects of biopolymer-based composites.

#### 1.1.3 Bionanocomposites: Pioneering Sustainable Solutions with Practical Significance

Nanocomposites have emerged as highly promising materials in recent years for a wide range of technical applications. These materials consist of multiple phases, with one component being at the nanodimensions. In the case of bionanocomposites, the matrix is typically a biopolymer, with fillers including metal oxides, clay, sand, carbon, and more. Biocomposites benefit synergistically from both components: the biopolymer contributes flexibility and strength, while the incorporation of nanoscale components imparts several exceptional characteristics to these composites, including a high aspect ratio, large surface area, excellent surface reactivity, improved functional density, high mechanical strength, and scaled possibility.^136,137^ The unique properties of biocomposites, including biodegradability, excellent strength, low cost, low density, suitability for physical and chemical modifications, easy separation, and environmentally friendly disposal, have propelled significant advancements in research across diverse fields, notably in water purification.^127,138−140^

The wide range of nanocomposites’ applications stems from the diverse structures and physical properties of the biopolymeric component.^141,142^ To achieve the desired performance, inorganic solids are often modified with biopolymeric layers, highlighting the crucial role of composite materials’ surface structure and behavior in polymeric properties.^143^ Examples of biocomposites include combinations of polysaccharides, like starch, cellulose, pectin, and chitosan, with inorganic solids such as silica, alumina, titania, zirconia, and iron in binary and ternary structures. These composite materials offer advantages such as porosity and high surface area. Biopolymers in composites exhibit improved efficiency, along with the possibility of metal oxides recyclability, which is not feasible when used alone or bare. However, separating spent nanomaterial can be challenging and costly. The subsequent part of this Review explores various nanocomposites comprising different metal oxides or binary oxides with various surface modifications. In particular, this Review highlights studies on chitosan/cellulose-based composites, selected for their practical relevance. This knowledge transfer in the field could guide future endeavors toward establishing low-cost media for commercial water treatment, specifically focusing on arsenic removal.

## 2 Chitosan-Based Nanocomposites: Versatile Adsorbents for Arsenic Removal

This part of this Review highlights progress in the structure of chitosan composites concerning their interaction sites, size, shape, adsorption ability for As(III or V), and application dynamicity. Chitosan or its functionalized derivatives serve as the base matrix, while metal oxides are predominantly utilized as fillers in nanodimensions. The array of fillers choices includes metal oxides, polymer, clay, silica, and other.

### 2.1 Enhanced Efficiency in Removing Either As(III) or As(V)

Chitosan in bead structure, impregnated with α-Fe_2_O_3_, maintains a maximum adsorbent capacity of 6.18 mg/g for As(III).^144^ Transforming iron-impregnated chitosan into a granulated structure further enhances its adsorption capacity to 6.48 mg/g at 1.0 ppm.^145^ Granular media with iron oxide particles ranging from 0.8–2.0 mm in packed bed column systems offer effective adsorption for practical reference.^146^ Thus, optimizing granulation is vital, particularly for the commercial application of composites in water treatment. Additionally, the critical aspect of the shape regulation lies in the potential for regeneration, where only a marginal regeneration loss of 20% has been observed even after 10 successive adsorption–desorption cycles.^144^

Integrating graphene oxide (GO) into magnetic chitosan-based nanocomposites (CMGO) offers an attractive means to introduce essential GO characteristics, such as an enhanced surface area (of 152.38 m^2^/g), resulting in increased adsorption to 45 mg/g for As(III) even at near-neutral pH 7.3.^147^ GO possesses diverse functional groups on its surface and edges, including epoxy, lactol, carboxyl, phenol, hydroxyl groups, and large π-stacking groups, contributing to high adsorption capacity through strong interactions like hydrogen bonding, electrostatic, and π–π interactions. Chitosan–magnetic graphene oxide grafted with polyaniline and doped with cobalt oxides offers appropriate acid/base responsive groups on the surface, enhancing the potential for higher removal. Indeed, the aforementioned study has revealed a removal potential of 89% within 50 min at a favorable neutral pH 7.0.^148^ Thus, the appropriate choice of components in a composite is crucial for practical relevance and commercial viability, as GO and polyaniline components are known for enhanced stability and cost-effectiveness, making them preferable for commercial applications.

**Figure 6:**
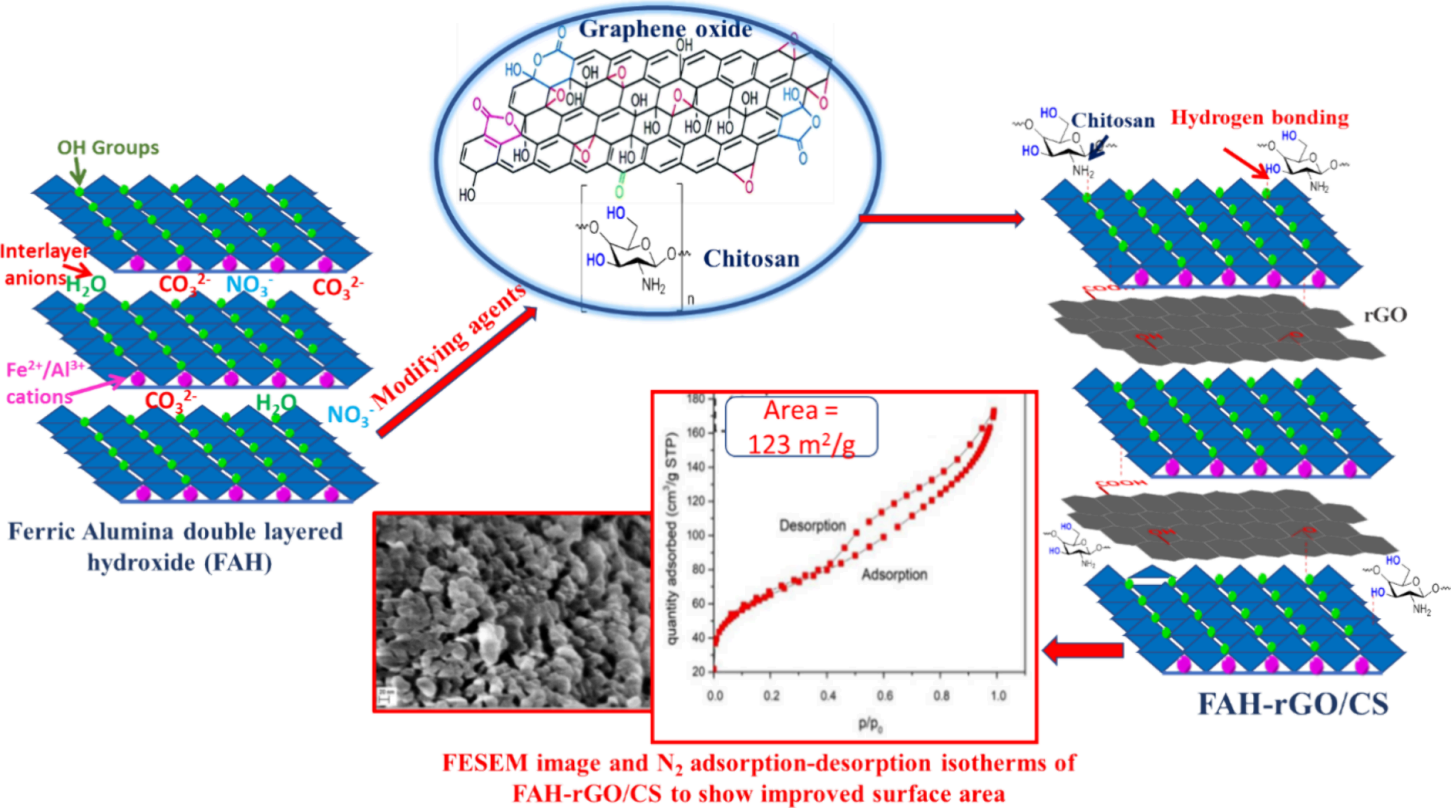
Schematic representing the intercalations of chitosan and FAH to the surface of rGO, FESEM image of FAH-rGO/CS, and improved surface area in FAH-rGO/CS. Reproduced or adapted with permission from ref (149). Copyright 2020 Elsevier.

The effective intercalation of chitosan (CS) and Fe–Al double-layered hydroxide (FAH, LDH) onto reduced graphene oxide (rGO) surfaces within the FAH-rGO/CS nanocomposite exposes the layers of rGO, thereby significantly increasing the surface area. This enhancement facilitates a remarkable removal of 97% and a maximum adsorption capacity of 167.79 mg/g for As(V) (as illustrated in Figure 6).^149^ Indeed, iron-based layered double hydroxides (LDHs), characterized by stacked layers with a positive charge and anions in the interlayer region, further endow a composite with an additional mechanistic capability to act as arsenic anion exchangers.

**Figure 7:**
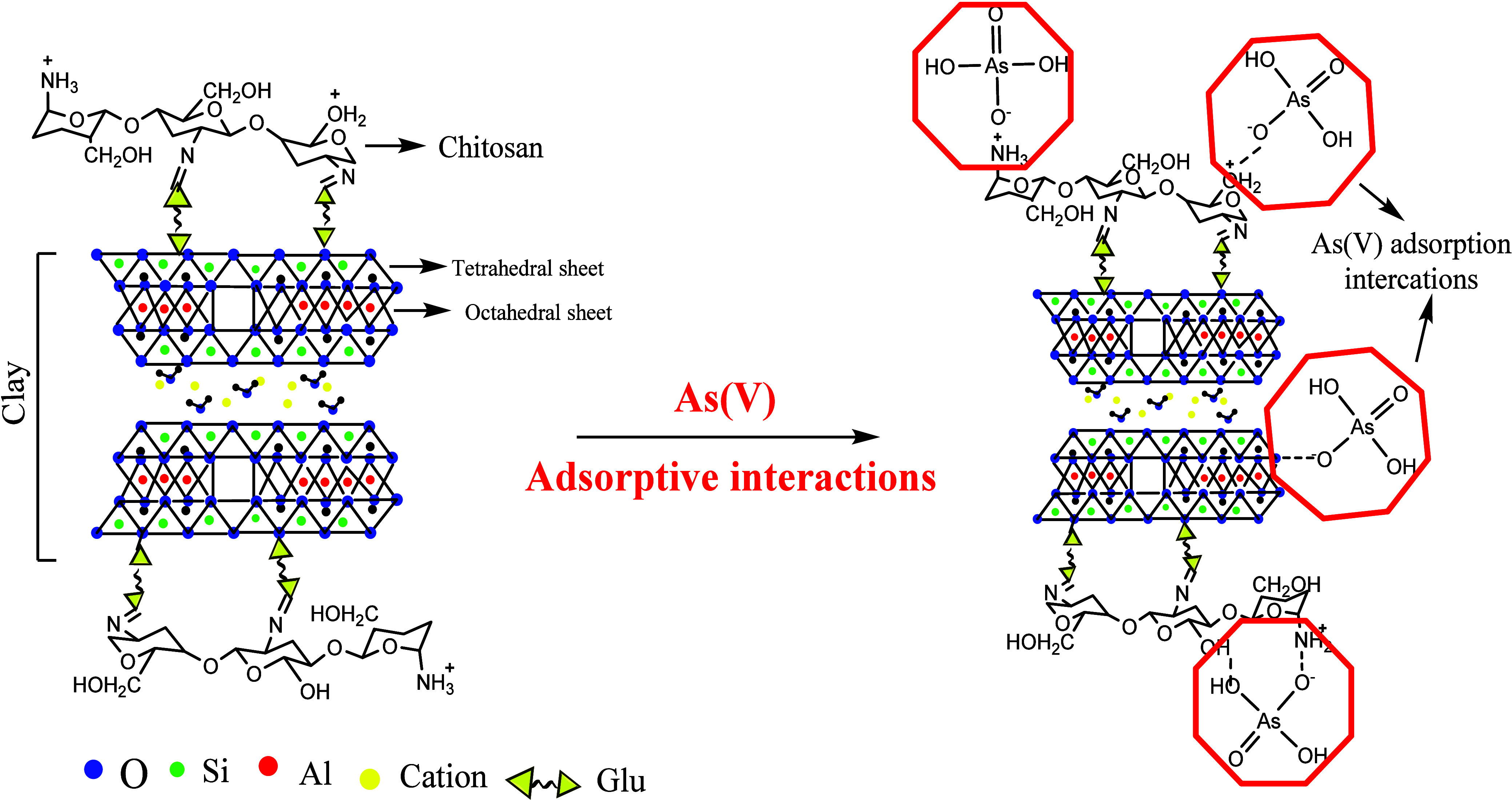
Schematic representing the structure of MMT/chitosan/Glu (glutaraldehyde) and probable different interactions in the adsorption mechanism of As(V). Reproduced or adapted with permission from ref (151). Copyright 2016 Elsevier.

Thus, multimechanistic possibilities in adsorption offer dynamic applicability in multicomponents systems, along with the improved adsorption capacity. Natural clays and waste fly ash, being low-cost components in composites, exhibit promising multimechanistic possibilities for adsorbing neutral, cationic, and anionic pollutants, as the structure of montmorillonite (MMT) clay is comprised of randomly oriented tetrahedral [SiO_4_]^4–^ and octahedral [AlO_3_(OH)_3_]^6–^ expandable layers, along with the presence of interlayer exchangeable ions like Ca, Na, and K to further enhance effectiveness in adsorption applications. For instance, studies on waste fly ash^150^/montmorillonite (MMT) clay-integrated-chitosan nanocomposites^151^ have demonstrated multiple electrostatic adsorption attraction sites for arsenate adsorption, including −NH_2_ functionality, Al^3+^ (Lewis acid), and AlOH^2+^/H^+^ (Bronsted acid sites) for As(V), even across various pH levels (Figure 7).

**Figure 8:**
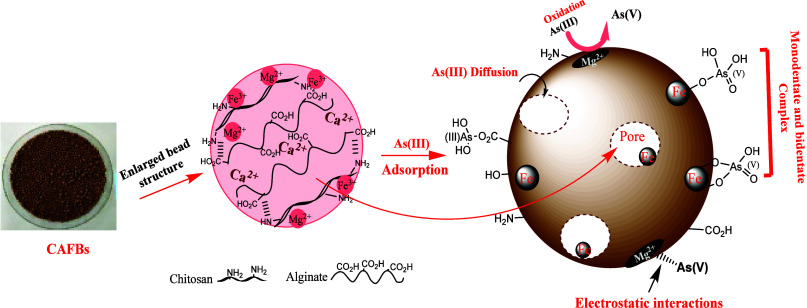
Multidynamic approach in As(III) adsorption on the surface of CAFBs. Reproduced or adapted with permission from ref (152). Copyright 2020 Elsevier.

The chitosan–alginate hybrid with manganese sludge (CAFBs) in bead structure also demonstrates dynamism in the adsorption process. When modified with manganese, it exhibits improved As(III) adsorption through cooperative control of redox mechanisms (where manganese sludge acts as an oxidant), complexation, diffusion, and electrostatic interaction (Figure 8).^152^ The significance of CAFBs lies in the fact that one of its components, manganese sludge, is a low-cost material. The resulting adsorbent, based on double metal oxides, possesses characteristics such as large surface area, abundant pores, strong adsorption affinity, a high density of active sites, redox activity, and mechanical stability. These attributes contribute to the effectiveness of the adsorption process, ensuring comprehensive pollutant removal.

While a multimechanistic approach may indeed be suitable for dynamic applications, a strong affinity is crucial to ensure the applicability of the adsorbent at trace levels. This becomes a major concern for practical relevance. Indeed, well-dispersed ultrafine cerium (Ce)-nanoparticles on the chitosan matrix effectively remove As(III) even at a concentration as low as 100 μg L^–1^ within 10 min.^153^ This involves monodentate and bidentate complexation of As(III) with surface −OH groups. Additionally, Ce(IV) oxidizes As(III) to As(V), adding to the redox mechanism of adsorption. However, regeneration of these adsorbents involving redox mechanism poses a challenge, as common acid/base-based regenerating agents lack the capability to convert Mn^2+^ to MnO_2_, which is crucial for restoring the oxidizing ability of these adsorbents.^152^ However, cerium-loaded chitosan/poly(vinyl alcohol) nanocomposites in nanofiber structure (CeCHT/PVA), a sophisticated physical modification, offer removal of As(III) well below the WHO-prescribed limit even in the absence of oxidizing agent, which is typically required as a pretreatment in removal of As(III).^154^ In fact, nanofiber morphology exhibits enhanced surface interactions due to the abundance of active groups on the surface, resulting in rapid adsorption. For instance, more than 90% adsorption for As(V) has been reported with amorphous and porous iron-functionalized chitosan nanofiber (ICS-ENF) within 100 min.^155^ Besides, nanofibers also prove a reduction in arsenate concentrations to the WHO limit of 10 μg/L in the effluent, even in a column adsorption setup with a feed solution consisting of As(V) and other coexisting anions.^156^

Indeed, the aforementioned studies conclusively demonstrate the improved capacities of nanocomposites for arsenic removal. However, an important aspect of nanocomposites is their stability in water systems, a vital prerequisite for actual application. In fact, robust stability can be achieved using the coprecipitation method with controlled alkalization in composite synthesis. For instance, chitosan–copper composites exhibit robust stability, preventing copper release with a remarkable recovery rate of over 95% even after undergoing five successive adsorption–desorption cycles.^157^

### 2.2 Scaling Up Chitosan Nanocomposites for Total Inorganic Arsenic (As III and V) Removal

Chemisorption stands out as one of the most operational approaches for comprehensive arsenic removal. However, adsorbing As(III) is challenging as it remains neutral across a wide pH range and requires oxidation to As(V) before adsorption. Thus, complete removal of total inorganic arsenic simultaneously poses a challenge due to the distinct properties of As(III) and As(V) in groundwater.

Iron(oxyhydr)oxide, a naturally occurring mineral, possesses strong affinity for both As(III) and As(V) over a wide pH range and is utilized in some current arsenic removal technologies.^158^ However, bare iron-based nanoparticles suffer from poor hydraulic properties and tend to aggregate during application. Zerovalent iron (ZVI) transformed into ZVI-chitosan nanoparticles (CIN) offers a wider pH range application. ZVI present in the adsorbent can reduce As(V) to As(III), which subsequently complexes with oxidized iron and chitosan. CIN also exhibits a high adsorption capacity, particularly 94 mg/g for As(III) and 119 mg/g for As(V) at pH 7.^159^ Precise selection of adsorbent components in a nanostructure is crucial for total inorganic arsenic removal applications in a single-step treatment option. In addition, the encapsulation of nanoparticles within polymeric structures ensures the efficiency of these mobile and reactive nanoparticles.

**Figure 9:**
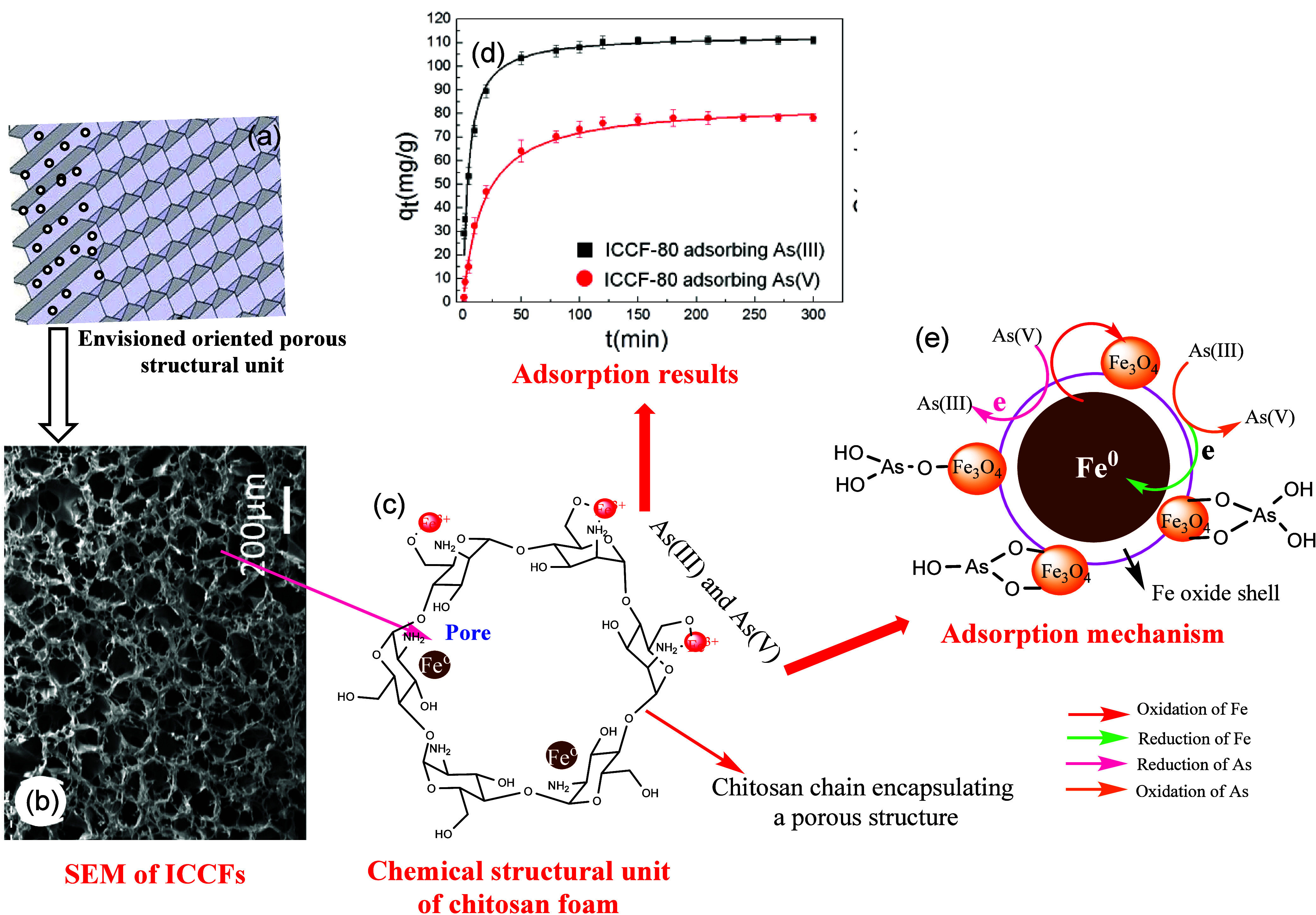
Schematic representation presenting a cross-sectional SEM micrograph of three-dimensional honeycomb-like structured zerovalent iron (nZVI)/chitosan composite foams (ICCFs) (a and b), its enlarged chemical structural unit around the pore (c), and arsenic interaction/adsorption results (d and e). Reproduced or adapted with permission from ref (160). Copyright 2016 Elsevier.

The three-dimensional (3D) honeycomb-like structures of nZVIs/chitosan composite foams (ICCFs) demonstrate exceptional removal efficiencies of 114.9 mg/g for As(III) and 86.87 mg/g for As(V) [at 200 ppm] due to the oriented porous 3D structure.^160^ Such comprehensive removal in ICCFs occurs through an adsorption-coupled reduction mechanism, leveraging the advantages of specialized morphologies, which also contribute to exceptional adsorption efficiency. Initially As(V) is reduced by adjacent nZVI, followed by the formation of a Fe^3+^–chitosan complex from the oxidized Fe^3+^ ion of nZVI. This process creates new active sites for the adsorption of additional arsenic ions on the nZVI–chitosan composite. Consequently, all arsenic species, including unreacted As(V), and reduced As(III) and As(0), can be adsorbed onto the ICCFs, offering a potentially practical solution to prevent the generation of secondary pollution from nZVI-associated species in water (refer to Figure 9).

**Figure 10:**
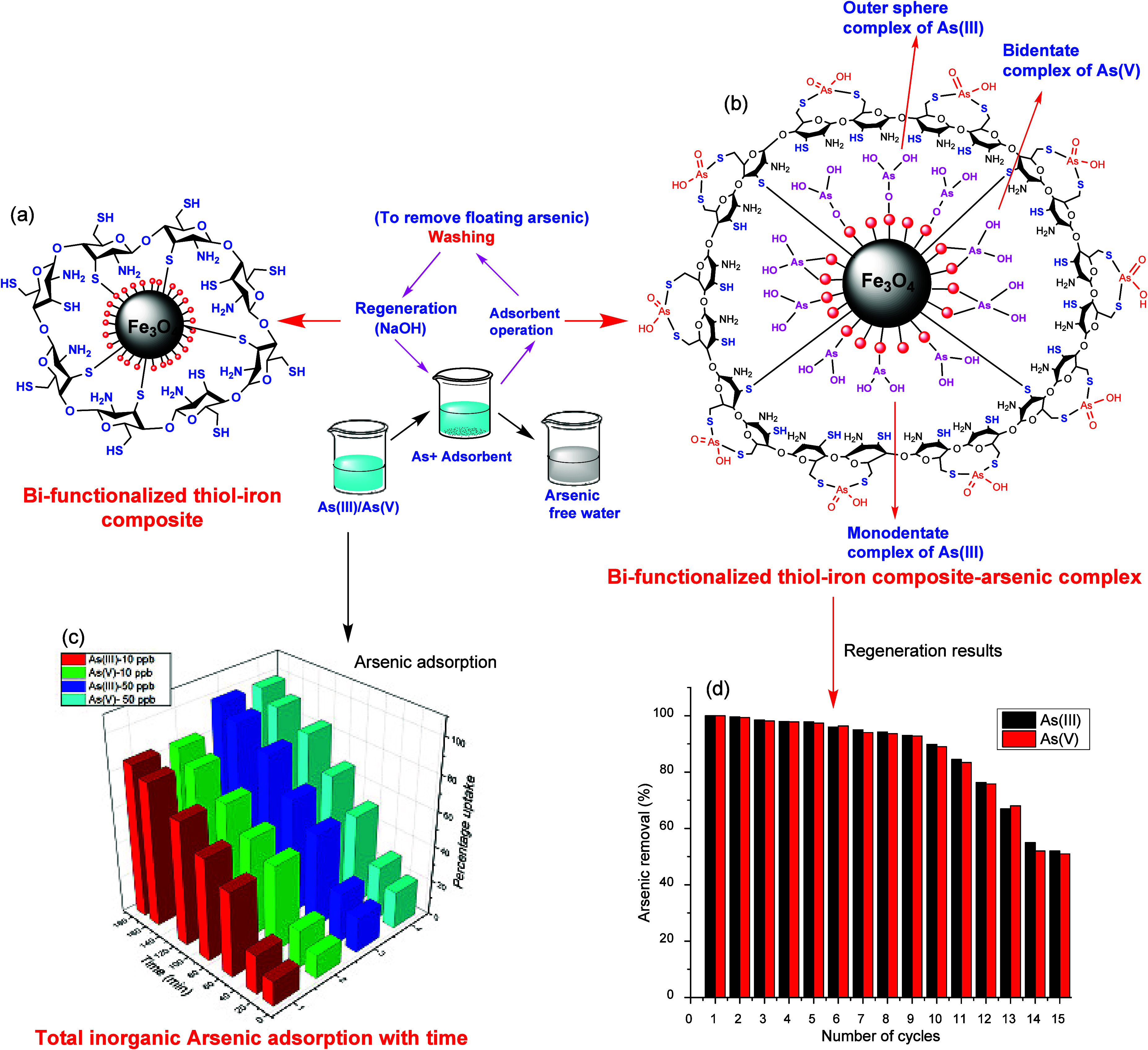
Structural unit of bifunctionalized TH-Fe composite (a), its different interactions in the adsorption process between composite and arsenic (b), and adsorption results (c and d). Reproduced or adapted with permission from ref (161). Copyright 2018 American Chemical Society.

The integration of strong affinity groups in a bifunctionalized chitosan–thiomer–Fe composite proves complete removal of total inorganic arsenic (over 99% removal of total inorganic arsenic) in just 2.5 h, even at a concentration as low as 50 ppb (Figure 10). This outstanding performance is attributed to the strong affinity of the −SH group for total inorganic arsenic, a challenging achievement, especially for both arsenic species As (III and V).^161^

**Figure 11:**
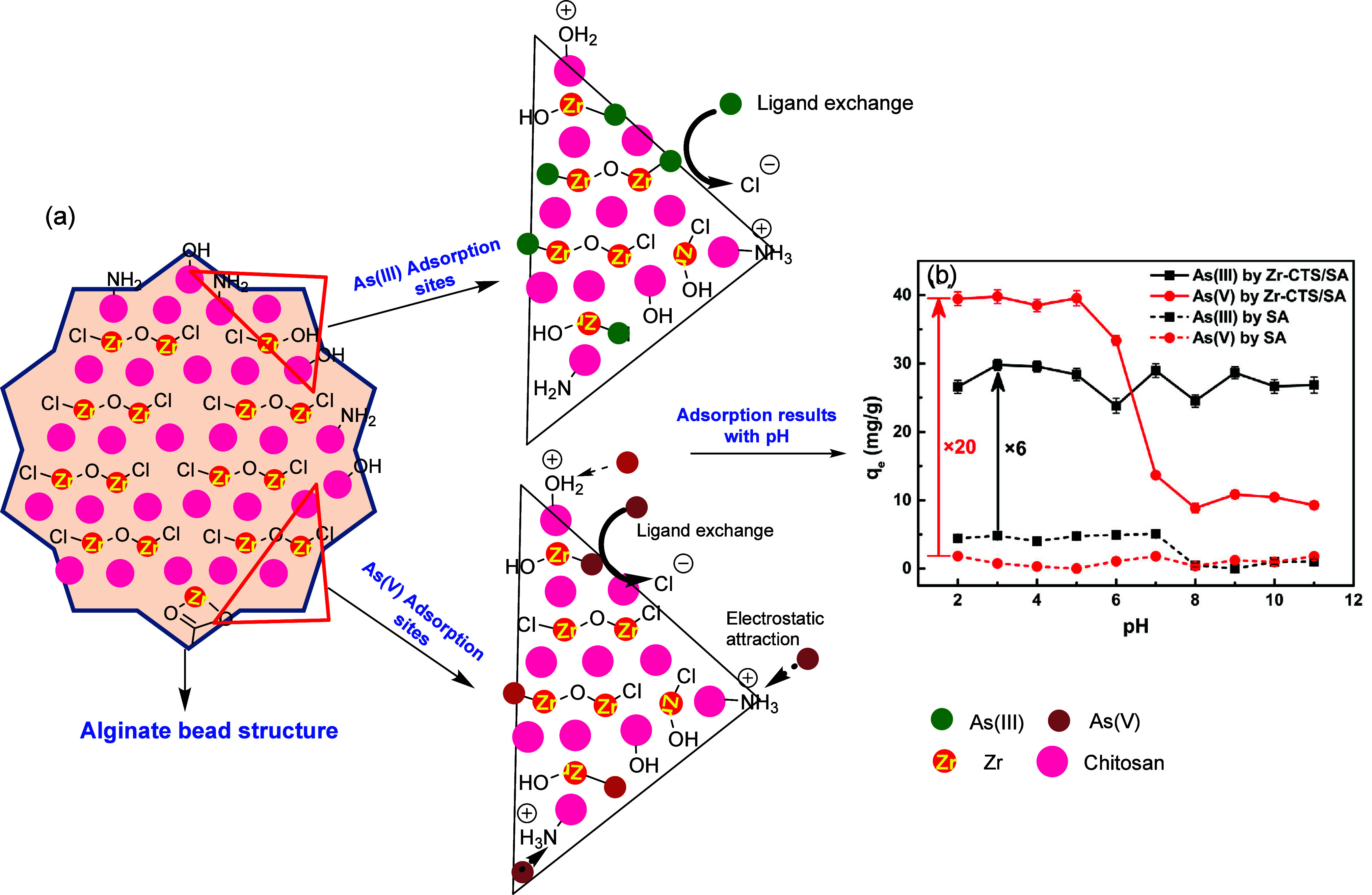
Schematic representing the adsorptive interactions of As(III/V) with zirconium-chitosan modified sodium alginate composite (a) and the adsorption results with pH (b). Reproduced or adapted with permission from ref (162). Copyright 2021 Elsevier.

The dynamics of adsorbent can further be increased by integrating acid/base sensitive groups. The −NH_2_/COOH as acid–base sensitive groups in zirconium-chitosan modified sodium alginate (Zr-CTS/SA) composite demonstrates minimal pH dependency on the removal of As(III). Specifically, the protonated −NH_3_^+^ and −OH_2_^+^ groups on the Zr-CTS/SA at low pH levels provide electrostatic attractions for negatively charged H_2_AsO_4_^–^ or HAsO_4_^2–^ ions during the adsorption process, as depicted in the pH-dependent adsorption results in Figure 11. Meanwhile, the adsorption of uncharged H_3_AsO_3_ As(III) occurs through Zr–O–As covalent bonds.^162^Figure 11 underscores the significance of the components structure through improved adsorption outcomes of Zr-CTS/SA compared to SA, one of the components in the composite, demonstrating multiple-fold enhancements.

Additionally, beads, fabricated using the in situ salt precipitation method, ensure a better distribution of iron particles in iron oxyhydroxide chitosan beads (IICB), resulting in a uniform distribution of active interaction sites. This contributes to higher arsenic removal efficiency via ion-exchange/inner sphere complexation compared to commercially available granulated ferric oxide/hydroxide-based adsorbents, that is, Bayoxide E 33 (12.85 mg As/g) and granulated ferric hydroxide (8.0 mg As/g GFH).^163^ Similar conclusions have been reinforced with chitosan goethite bionanocomposites (CGB) beads, demonstrating efficiency in treating synthetic arsenic water to WHO acceptable standards, validating practical application possibilities.^164^

Recent advancements involve evolving from single metal–oxide designs to binary oxides-based polymeric adsorbents for complete removal of As(III) and As(V) without the need for pretreatment. Components with a natural affinity for arsenic, such as Al_2_O_3_ and TiO_2_ nanoparticles, in mixed oxide-based adsorbents offer a higher removal efficiency in synergy,^165^ wherein Al_2_O_3_ exhibits a high affinity for As(V), while TiO_2_ can oxidize As(III) to As(V) upon exposure to UV. Manganese oxides have also been employed as oxidizing agent in mixed metal oxides-based adsorbents for total inorganic arsenic removal.^166^ In the evolution toward binary oxides, magnetic nanoparticles consisting of zirconium-Fe_3_O_4_@Zr(OH)_4_ impregnated into chitosan beads, referred to as magnetic nanoparticles impregnated chitosan beads (MICB), have shown comparable efficiency in removing both As(III and V) species without any pretreatment requirement, attributed to the strong binding affinity of zirconium for arsenic.^167^

Incorporating inexpensive materials, such as clay or sand alongside iron-chitosan, a well-evidenced composite for total arsenic removal, presents a promising avenue for the development of cost-effective technologies, particularly in rural areas. Iron-chitosan-coated sand columns have demonstrated a high breakthrough capacity for the simultaneous removal of total inorganic arsenic As(III and V).^168^ Notably, in one study, chitosan composites with confined metastable 2-line ferrihydrite, an iron oxide mineral, integrated into domestic water filtration units, consistently provided clean water at a rate of 6000 L per year, with arsenic levels consistently below the WHO limit of 10 ppm. Furthermore, the system’s simplicity, utilization of readily available raw materials, and autonomy from electrical power sources render it highly accessible. Its implementation in resource-limited settings could potentially offer arsenic-free drinking water for a family of five at an estimated annual cost as little as $2.^127^ The comparative adsorption capacities of chitosan-based composites for total inorganic arsenic removal under different pH conditions are presented in Table 2, highlighting the capability of only a few chitosan-based adsorbents to remove both species As(III and V), simultaneously without pretreatment under natural conditions.

**Table 2:** Comparative Adsorption Capacities of Chitosan-Based Composites for the Removal of Total Inorganic Arsenic with pH

		adsorption capacity (mg/g)		
adsorbent	pH	As(III)	As(V)	ref
chitosan	4.0		58.0	(169)
chitosan-coated alumina biosorbent	**4.0**	**56.5**	**96.5**	(170)
chitosan beads impregnated with iron (FICB)	8.0	6.48		(145)
iron-coated chitosan flakes (ICF)	**7.0**	**16.15**	**22.47**	(171)
chitosan–Fe-cross-linked complex	9.0	13.4		(172)
magnetic chitosan chelating resin	2.0	62.42		(173)
cross-linked chitosan and nanomagnetite	3.0–9.0	5.9		(174)
iron(III)-loaded chitosan hollow fiber membranes	<6.4	0.0037		(175)
chitosan beads immobilized with iron(III)	**7.0**	**21.24**	**27.59**	(176)
Al–Fe-chitosan	3–9	16.94		(177)
chitosan loaded with cerium (Ce-CHT/PVA)	6.2–7.0	18.0		(154)
α-Fe_2_O_3_, impregnated chitosan beads	5.0	6.18		(178)
As(III)-imprinted chitosan resin (As-ICR)	6.0	4.16		(179)
iron–chitosan composite granules	7.0	16.15		(180)
granular adsorbent (GA)	6.5		14.95	(146)
zerovalent iron encapsulated chitosan nanospheres (ZIEN)	**7.0**	**94**	**119**	(159)
iron–chitosan-coated sand (ICCS)	**7.0**	**26**	**56**	(155)
lanthanum immobilized electrospun chitosan nanofiber (CSN-La)	4.0–6.0		83.6	(156)
chitosan-coated Na–X zeolite	2.1		63.23	(150)
cerium modified chitosan ultrafine nanobiosorbent (Ce-CNB)	8.0	57.5		(153)
iron-functionalized chitosan electrospun nanofiber (ICS-ENF)	7.2		11.2	(168)
chitosan-based magnetic hybrid material	6.0–8.0		147	(181)
Fe–Mn binary oxide impregnated chitosan bead (FMCB)	**7.0**	**54.2**	**39.1**	(166)
chitosan-goethite bionanocomposite (CGB)	5.0–9.0	**8.5**	**11.3**	(164)
SiO_2_@hybrid chitosan beads			1.69	(182)
magnetic nanoparticles impregnated chitosan beads (MICB)	6.8 at 50 ppm	**35.3**	**35.7**	(167)
	10 ppb	3.17	4.02	
nZVI/chitosan composite	4.0–7.0	**114.9**	**86.87**	(160)
chitosan-thiomer	6.0–8.0	**17.08**	**17.66**	(120)
chitosan-based MCS/ZnO@Alg gel microspheres	6.0		63.69	(147)
FAH-rGO/CS nanocomposite	5.0–7.0		167.79	(149)
chitosan-entrapped zirconium (Chi–Zr) biocomposite	5.0		190	(183)
protonated chitosan flakes (PCF)	6.0		5.43	(184)
chitosan-graphene oxide-gadolinium oxide (CGO-Gd) nanocomposite	3.0–7.0		252.12	(185)
chitosan-iron oxide-graphene oxide composite beads (GO-BDs)	8.0	0.081		(186)
steel slag recovered iron–chitosan composite	4.0		11.76	(187)
sustainable magnetic chitosan biosorbent beads	**6.7**	**73.69**	**79.9**	(188)
bifunctional thiolated-chitosan-iron composite	**7.0**	**91**	**88**	(161)
Zr-CTS/SA		**43.19**	**76.8**	(162)

## 3 Advancements in Cellulose-Based Composites: Versatile Adsorbent for Arsenic Removal

Cellulose, the most abundant organic compound on earth, offers itself as an excellent choice as a matrix in nanocomposite structure for arsenic adsorption, particularly for practical applications, owing to its superior mechanical strength. The potential and selectivity of an adsorbent primarily depend on its chelating groups, which can be enhanced in cellulose due to its amicability for functionalization of −OH groups at the C2, C3, and C6 positions. Building upon this, the subsequent part of this Review sheds light on the advancements in cellulose-based composite structures for arsenic removal.

### 3.1 Specific Removal of Either As(III) or As(V) Using Cellulose-Based Composites

Simultaneous and complete removal of arsenic is challenging, as arsenic exists in various species depending on the pH. However, cellulose composites, such as cellulose-metal oxide/hydroxide adsorbents, feature a variety of functionalities like −OH, OH_2_^+^, and −O^–^, as interaction sites for adsorption, the chemical nature and abundance of which are influenced by the solution’s pH. For example, in acidic pH, OH_2_^+^ groups predominate and provide electrostatic attraction as the governing adsorption mechanism between As(V) and positively charged sites on the nanocomposite’s surface.^189,190^ Indeed, As(V) predominantly exists as H_2_AsO_4_^–^ between pH 2 and 6.

**Figure 12:**
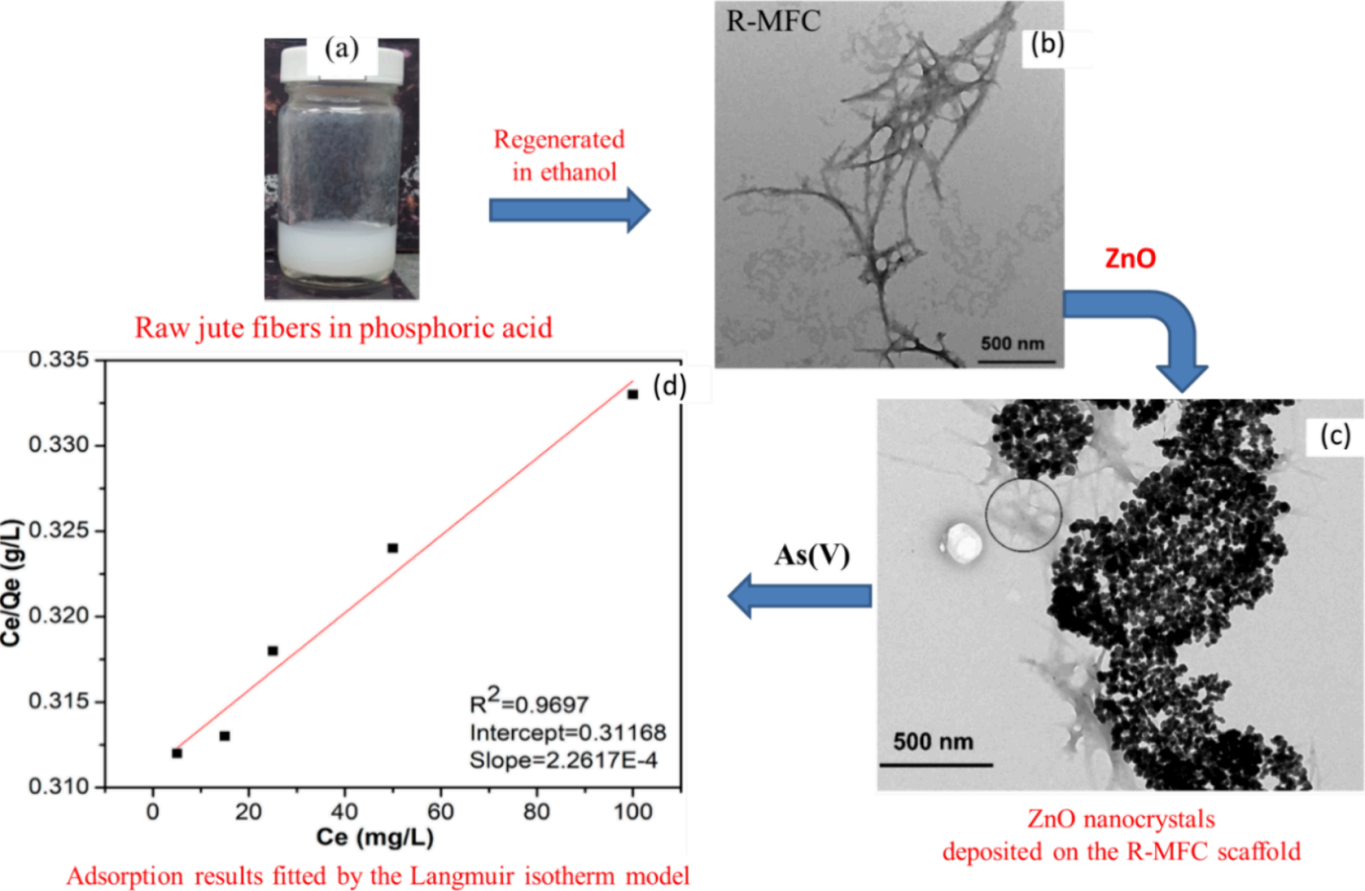
Schematics representing the regeneration process (a), TEM images of R-MFC and ZnO-decorated-R-MFC (b and c), and As(V) adsorption results with concentration (d). Reproduced or adapted with permission from ref (191). Copyright 2019 American Chemical Society.

As the effectiveness of an adsorbent relies on the number of interaction sites available, controlled morphology in cellulose structure emerges as a promising approach to enhance the capacity and mechanical strength of a nanocomposite. The regeneration process offers a uniform structure of fibers on the nanoscale. For instance, raw jute fibers, when regenerated from a phosphoric acid solution with ethanol, result in microfibrillated cellulose (R-MFC) on a nanometer scale, which can be used as a scaffold. Incorporating zinc oxide crystal (ZnO/R-MFC) onto this scaffold leads to significant structure modification across the fiber, as evidenced by high density anchoring of ZnO in TEM images in Figure 12.^191^ Consequent to this dense modification, the ZnO/R-MFC composite exhibits a maximum adsorption capacity of 4421 mg/g according to the Langmuir isotherm model within the concentration range of 5.0–100 ppm.

**Figure 13:**
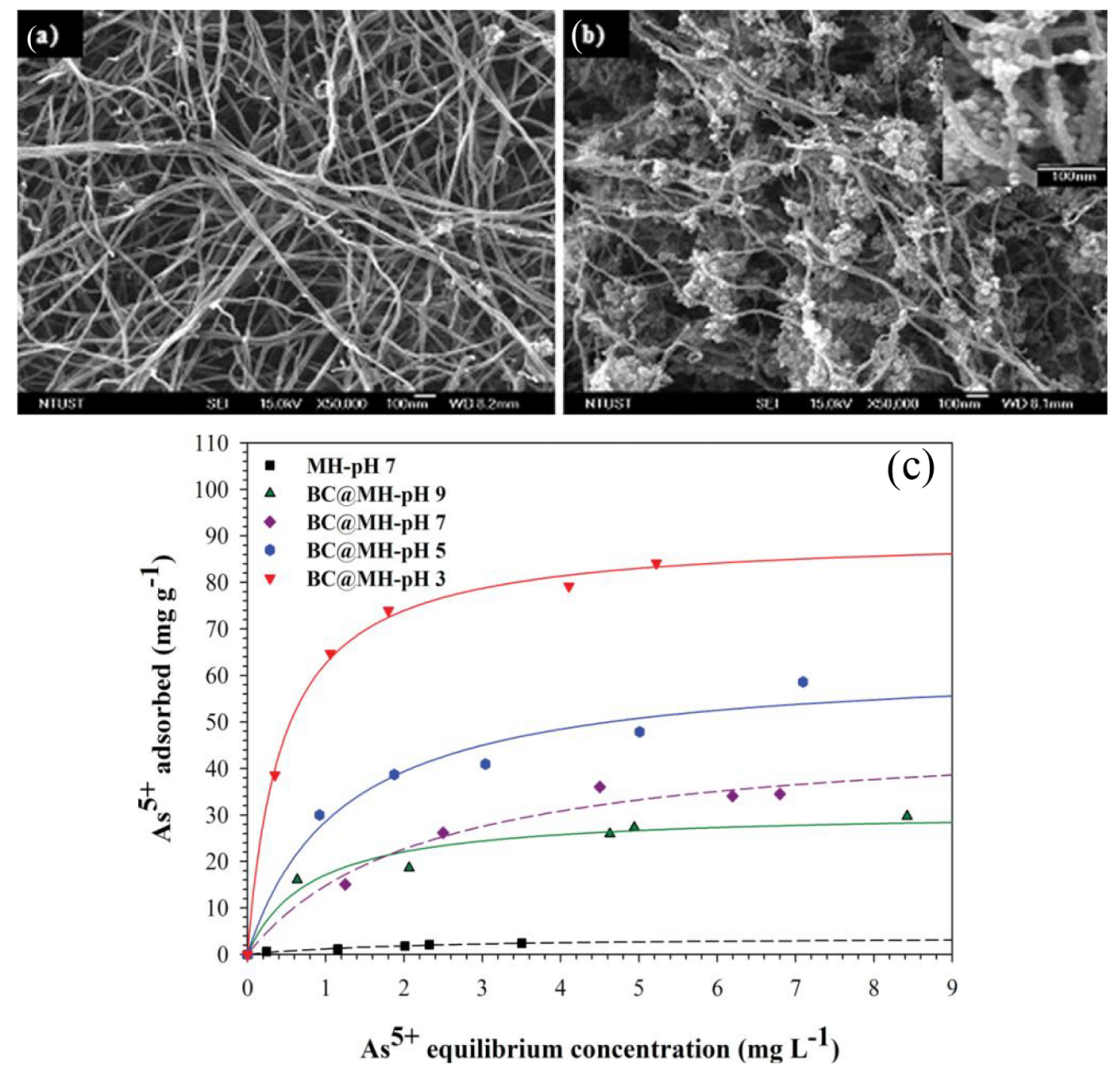
FE-SEM image showing the finer structure of (a) bacterial cellulose, (b) top view of BC@MH, and (c) adsorption isotherm of As(V) onto BC@MH and MH (amine-functionalized MNPs prepared in the absence of BC pellicle). Reproduced or adapted with permission from ref (192). Copyright 2011 Royal Society of Chemistry.

Furthermore, the nanofibril derived from bacterial cellulose (BC), which possesses an exceptionally small dimension width of 20–30 nm, 200-times finer than plant cellulose, when functionalized with aminated-magnetite nanoparticles (MH) exhibits a maximum adsorption capacity of 90 mg/g, even at trace concentrations as low as 7.0 ppm. The reduced size of the nanofibrils theoretically results in an increased surface area in BC, characteristics supported by SEM images depicting ribbon-shaped porous structures interconnected in a three-dimensional arrangement. The augmented surface area of BC nanoparticles provides numerous nucleation sites for aminated-ferric ions to undergo magnetite growth, as observed in granular formulations along the length of the fibrils in Figure 13 of BC@MH.^192^ The presence of aminated-magnetite nanoparticles in BC@MH also confirms sensitivity to arsenic at trace levels, a feat often challenging to achieve without the high density of aminated nanoparticles.

Intercalating carboxymethyl cellulose (CE) into the interlayer space of Fe–Al/reduced graphene oxide (FAH-rGO/CE) nanocomposites can be adjusted to modulate adsorption properties, resulting in a significant enhancement in As(V) adsorption (up to 98%) with increasing weight percentage of CE. The intercalation of CE deforms the flake-like hexagonal structure of FAH-rGO composites, creating a surface roughness that activates adsorption sites.^193^ Moreover, iron-based layered double hydroxide (LDH) has recently gained significant attention as a key component in adsorbents owing to its unique physicochemical properties, including rapid regeneration, high surface area, high capacity, and tunable properties.

While cellulose-based adsorbents have been explored for As(III) removal, only a few studies support its sorption due to the difficulty in removing this species, given its nonionic nature. As As(III) requires a strong-affinity group for adsorption, dithiocarbamate-cellulose-based silica composite (DTC) removes As(III) by more than 80% over a broader pH range, as arsenic is thiophilic.^194^

In the absence of strong-affinity groups, oxidizing agents are required in nanocomposite to remove As(III) from aqueous systems. For instance, a composite (FeOOH/CuO@WBC) derived from bamboo cellulose (WBC, a wood bamboo cellulose) with components such as CuO and FeOOH helps in As(III) adsorption by transforming it to As(V).^195^ Additionally, a composite of clay with cellulose, specifically hydroxyapatite-bentonite clay-nanocrystalline cellulose (CHA-BENT-NCC), has demonstrated over 95% removal of As(III) in adsorption kinetics equilibrium within 5 min, even at 50 ppm.^196^ The rapid kinetics is attributed to the choice of components in the composites, where clays serve as excellent adsorbents due to their high specific surface area, layered structure, and high cation exchange capacity. Simultaneously, nanocrystalline cellulose acts as a template to disperse hydroxyapatite (CHA) and clay mineral particles in the cellulose matrix with uniform morphology, thereby enhancing kinetics.

### 3.2 Scaling Up Cellulose-Nanocomposites for Total Inorganic Arsenic (As III and V) Removal

As emphasized earlier, the structure of the nanocomposite plays a crucial role in reference to the size, shape, and nature of functional groups for achieving complete and maximum adsorption capacity. The one-step coprecipitation method proves to be an effective approach in achieving a high specific surface area of 113 m^2^ g^–1^ for cellulose-based-composites, significantly higher than that of pristine cotton structures (1.081 m^2^ g^–1^), thereby enhancing total arsenic removal.^197^ Indeed, carboxymethyl cellulose-ferrihydrite (CMCFH), containing a functionalized cellulose matrix in a cartridge setup, produces output water with total arsenic levels well below the WHO permissible limits, with arsenic concentrations as low as 200 ppb, which can be attributed to enhanced interaction points.^198^

However, nanoscale zerovalent iron (NZVI), due to its strong reducing property and small size, is prone to passivation and loss of adsorption capacity. In NZVI@SiO_2_@cellulose (FCS) composites, the porous silica offers a solution to alleviate NZVI’s oxidation problem. The adsorption mechanism in FCS involves an oxidation–reduction reaction wherein NZVI converts toxic oxidized arsenic into nontoxic elemental arsenic.^199^ The thin layer of silicon dioxide with particle sizes ranging from 10 to 20 nm effectively protects the adsorption capacity of NZVI embedded within the cellulose matrix.

Given the presence of different arsenic species in water, achieving total removal can be challenging. However, the intercalation of LDH (Zn/Al) with cellulose, known as Zn/Al CZA, has demonstrated comparable removal potential for both arsenic (III and V), even at pH 6.0.^200,201^ Moreover, the removal of As(III) occurs via a one-step mechanism, unlike other methods that involve a two-step process of oxidation to As(V) followed by adsorption. Table 3 presents the comparative adsorption capacities of cellulose-based composites for arsenic removal with respect to pH. Unlike chitosan, achieving the removal of total inorganic arsenic is more challenging with cellulose-based composites, with only a few composites capable of removing both species (data highlighted in bold font).

**Table 3:** Comparative Adsorption Capacities of Cellulose-Based Composites for Arsenic Removal with Respect to pH

		adsorption capacity (mg/g)		
adsorbent	pH	As(III)	As(V)	ref
rice husk	6.85	NA	≈0.0405	(202)
cellulose loaded with iron oxyhydroxide	**7.0**	**99.6**	**33.2**	(203)
functionalized microcellulose-reinforced 2-lineferrihydrite composite	4.0–10	**143**	**83**	(199)
amine-rich magnetite/bacterial cellulose-nanocomposite (BC@MH)	3.0		36.49	(192)
hydroxyapatite-bentonite clay-nanocrystalline cellulose	4.0–7.0		53.89	(196)
cellulose@iron oxide nanoparticles	6.0–9.0	**23.16**	**32.11**	(197)
cellulose@iron nanoparticles composites	8.0	92.95		(204)
ZnO/R-MFC	7.0		4421	(191)
NZVI@SiO2@celluloses (FSC)	2.8–4.0	70		(198)
cellulose-*g*-GMA-*b*-TEPA	**5.0–7.0**	**5.71**	**75.13**	(205)
microscale dialdehyde cellulose–cysteine (MDAC–cys)		344.82		(118)
nanoscale dialdehyde cellulose–cysteine (NDAC–cys)		357.14		
dithiocarbamate-modified cellulose resins (DMC-2)	3.0	47		(194)
cellulose-carbonated hydroxyapatite nanocomposites (CCHA)	7.0		12.72	(206)
Fe(III)-coordinated amino-functionalized poly(gycidylmetha-crylate)-grafted TiO_2_-densified cellulose (AM-Fe-PGDC)	6.0		105.47	(207)
modified microfibrillated cellulose (FeNP/MFC)	2.0		2.46 mmol/g	(190)
cellulose-based anion exchanger (Cell-AE)	6.0		99% removal	(208)
polyethylenimine (HPEI) modified cellulose fiber (cell_MW_-HPEI)	4.0–7.0	**54.13**	**99.35**	(24)
carboxymethyl cellulose- zerovalent iron-based composite	5.0–7.0	**12.2**	**14**	(209)
functionalized cellulose nanofibrils			25.5	(210)

## 4 Decoding the General Mechanisms of Arsenic Adsorption in Key Materials of Practical Significance

Arsenic contamination in water presents a significant challenge necessitating comprehensive remediation strategies. To optimize adsorption processes, a deeper understanding of the interaction between chitosan/cellulose and arsenic species is crucial. Relying solely on a single removal mechanism may prove inadequate due to the complexity of the environment and the varying forms of arsenic. Hence, adopting a multimechanistic approach, which utilizes different methods concurrently or sequentially, is essential for effective arsenic removal, targeting both inorganic forms in water.

This Review has demonstrated that both As(III) and As(V) interact with chitosan/cellulose-based adsorbents through mechanisms such as hydrogen bonding, electrostatic attraction, and/or chemical bonding. Metal nanoparticles, including iron and aluminum, facilitate covalent interactions between chitosan/cellulose and themselves through abundant functional groups, such as −O^–^, −OH, and NH_2_ on scaffold, ensuring the availability of charged sites on metal oxides for arsenic adsorption. Typically, metal oxide surfaces offer sites like MOH, MOH_2_^+^, and MO^–^, with their proportions determinant to the pH level. The arsenic adsorption mechanism involves the coordination of hydroxyl groups of iron hydro(oxides) with the OH^–^ ligands in the arsenic molecule. In this specific adsorption, the iron oxyhydroxide nanoparticles located on the biopolymeric surface are capable of replacing the OH^–^ ligand of arsenates’ molecules, forming mono- and bidentate complexes allowing them to be attached to the surface.^211,212^

**Figure 14:**
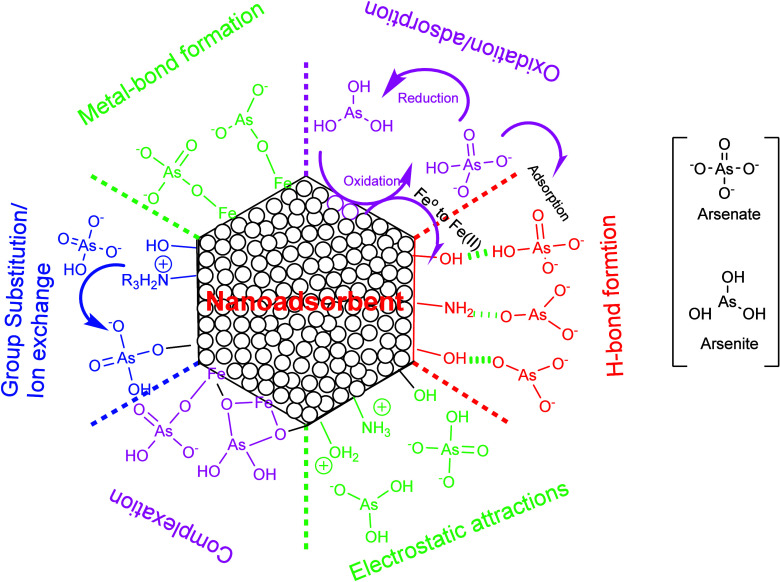
A potential graphical representation depicting dynamic mechanisms for As(III and V) adsorption in bionanocomposites, with iron nanoparticles as a reference case.

In iron nanoparticles-based nanocomposites, the redox reactions occurs in the presence of oxygen or other oxidizing agents, where Fe(0) is oxidized to Fe(II) or Fe(III) and arsenic species are reduced. This results in the formation of iron oxide/hydroxide layers on the nanoparticle surface, further enhancing the adsorption capacity for arsenic species through coordination interactions and ligand exchange with the iron oxide/hydroxide phases at the Fe(0) core–oxide shell structure of nZVI. Thus, As(III) and As(V) either complex or coordinate on the surface of metal oxides via the formation of bidentate and monodentate complexes, while negative arsenate can be adsorbed either through an exchange process with OH^–^ or by adhering to metal locations.^213^Figure 14 presents the possible dynamic mechanism with iron-biocomposites. In conclusion, by strategically integrating diverse removal mechanisms, the objective of achieving total arsenic removal from water sources can be effectively and sustainably realized. This approach addresses the complexity of arsenic contamination comprehensively and ensures the mitigation of this pressing environmental issue.

## 5 Reviving Strategies: Regeneration and Reuse of Biopolymer-Based Composites

Regenerating and reusing spent adsorbents is crucial for their practical application in commercial water treatment. The regeneration process must effectively restore the initial sorption capacity of the adsorbent. However, the efficiency of desorption depends on several factors, including the chemical and physical properties of the adsorbent, the characteristics of the eluents, and the presence of competitive ions in the aqueous environment.

**Figure 15:**
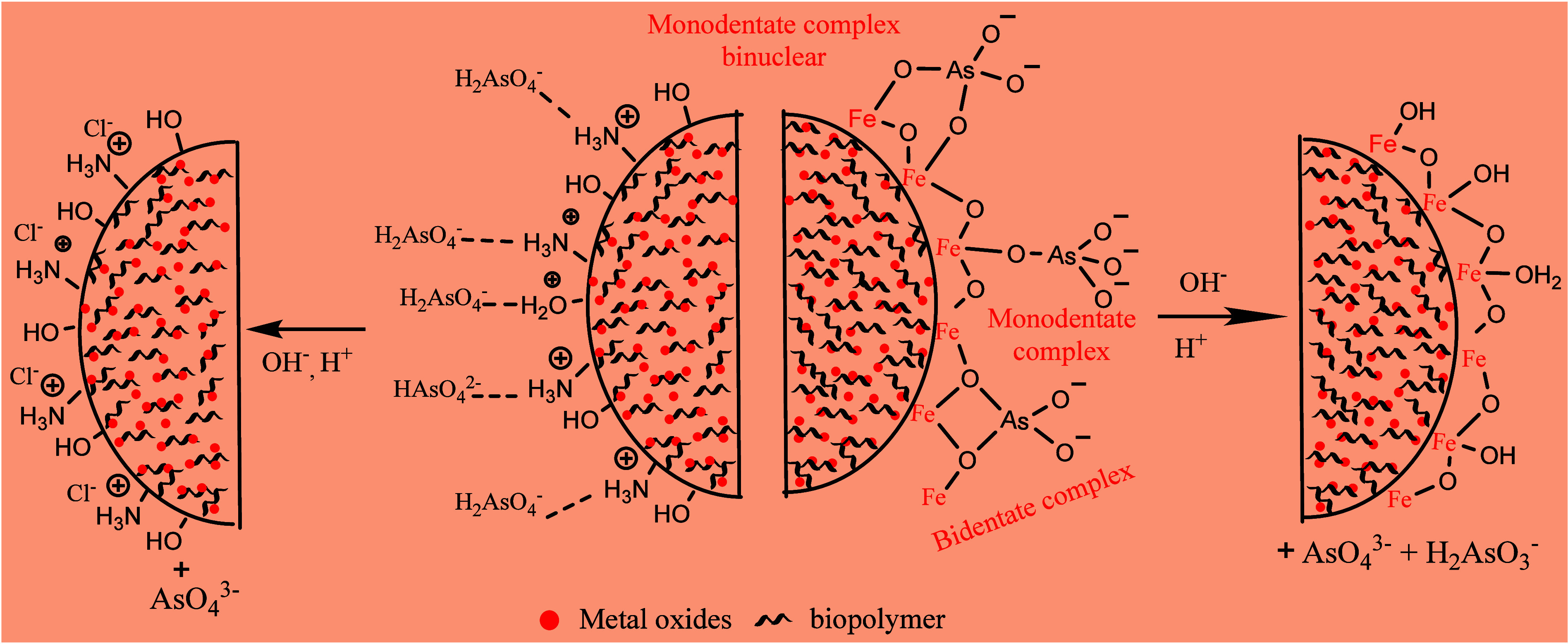
Possible mechanisms of arsenic [III and V] desorption, subsequent to adsorption through electrostatic and chemisorption mechanisms.

Typically, acidic and basic solutions are commonly used as desorption agents to remove arsenic from loaded composites due to the varying pH-dependent species of arsenic. The interactions between the adsorbent and arsenic are also subjective to pH variations. Basic pH leads to the deprotonation or neutrality of sorption sites, thereby facilitating adsorbent regeneration. With pH variation, surface groups change their forms and aid in the regeneration process. The mechanism is expressed in Figure 15.

Generally, higher eluent concentrations, such as 0.1 M NaOH solutions, have been used in adsorption–desorption studies with chitosan/cellulose nanocomposites.^129,159,214^ However, excessive eluent concentration may compromise the integrity of the filtration media. Conversely, lower concentrations of eluents also offer promising results, maintaining high desorption efficiency without a significant impact on composite properties.^161^ Indeed, in one study, desorption rates above 98% were reported in iron-chitosan nanofiber using 0.003 M NaOH solution, with no noticeable effect on color, weight, size, and shape of composites even after 10 consecutive adsorption–desorption cycles.^156^

The ease or efficiency in desorption depends upon the strength of coordination or mechanistic details. Therefore, a slight shift in solution pH will change the surface charge of materials removing weakly adsorbed As(V) molecules. At basic pH, the surface of the adsorbents is negatively charged, thereby promoting the rejection of negatively charged adsorbed molecules, while desorption of the −OH ligand interchange may be difficult to achieve.

Biopolymeric composites containing silica or clay components can also be effectively eluted using basic solutions with a pH of ≥11.^215^ In fact, the eluting solution of 0.01 M NaOH even shows only a marginal decrease in adsorption from 87% to 85% from the first to fourth adsorption–desorption cycles in chitosan–LDH composites.^216^

A practical adsorbent should exhibit both high removal efficiency and reusability potential, even in the presence of competing metal ions. Under weak acidic or neutral conditions, competitive anions such as phosphate and silicate species may interfere with arsenic adsorption. Conversely, at basic pH levels, dominant species like HPO_4_^2–^ and H_2_SiO_4_^2–^ facilitate desorption through competitive mechanisms.^217,218^ Nevertheless, future research should focus on exploring novel eluent methods and optimizing conditions to enhance the regeneration and reuse of biopolymer-based composites for commercial applications.

## 6 Conclusions and Future Prospects: Advancing Sustainable Water Treatment Technologies

In the 21st century, population growth and technological advancements have significantly increased the global demand for resources such as materials, energy, food, and water. Nanocomposites, particularly those incorporating biocomponents, are emerging as cost-effective solutions to enhance environmental sustainability. Addressing intricate issues like arsenic contamination in water remains a pressing concern, necessitating the development of affordable, user-friendly technologies to ensure safe drinking water.

In recent years, iron oxides or carbon impregnated with iron have been widely used for arsenic removal, but they face challenges related to agglomeration, cost, and safe disposal. Utilizing nanoparticles loaded onto support materials offers a promising solution. Biopolymers, notably cellulose and chitosan, stand out as economical and exceptional scaffold choices, providing versatility and high functional density for multimechanistic sustainable media across various technical applications. In adsorptive media systems, the operation and maintenance costs are substantial, with the cost of replacing adsorptive media accounting for approximately 80% of these expenses. Transitioning to alternative, lower-cost, and higher-performance media based on biopolymers could represent a future technology with widespread applications that can be readily implemented at the household or community levels.^219^

This Review underscores researchers’ endeavors in crafting nanostructured cellulose and chitosan materials to reinforce their adsorption capabilities, particularly for total arsenic removal. Nanostructuring amplifies specific surface area, yielding more active sites, thus enhancing overall efficacy. Moreover, this Review addresses crucial factors like methodological reproducibility stemming from the intricate nature of nanocomposite preparation and environmental ramifications, pivotal for assessing their potential at a large scale. Furthermore, this Review concludes that integration of various counterparts such as polymers, carbon materials, magnetic nanoparticles, clay, zeolites, and metal nanoparticles in binary and ternary structures enhances the capacities of chitosan/cellulose nanocomposites. Integrating multiple mechanisms enables the attainment of synergistic effects, resulting in enhanced arsenic removal efficiency and broader applicability across varying water compositions and contamination levels. Furthermore, a multimechanistic approach provides flexibility in system design, allowing for customization based on specific site requirements and treatment objectives. Thus, natural resources, such as clay and bentonite, can serve as viable replacements for transition metal-based composites for arsenic removal, being environmentally friendly and posing no risks to humans.

Nevertheless, despite promising advancements, significant research gaps persist. Critical among these gaps is the absence of pilot and field-scale applications of these materials, essential for validating laboratory performance under real conditions and enabling comprehensive technoeconomic analyses. Indeed, only a few studies confirm the viability of green-composites as alternative media in domestic water filtration units for complete arsenic removal for a family of five at an estimated annual cost of only $2.^127^ Additionally, detailed investigations into the environmental impact, long-term stability, and potential ecotoxicity of nanocomposites are imperative. Transitioning from batch tests to column tests would provide a more realistic assessment of material efficiency and performance in practical settings, addressing mechanical aspects such as pumping pressure, flow rate, and channeling effects, which are currently overlooked. Further research is warranted to apply nanocomposites in treating complex, multicontaminated waters.

Therefore, this Review categorically provides a better understanding of arsenic adsorption mechanisms, which can provide a rationale for the design and fabrication of new nanocomposites for practical relevance. We anticipate that researchers will soon develop innovative cellulose/chitosan-composites as scaffolds for viable technologies. Overcoming specific challenges, such as designing for practical applications and addressing stability issues, will be crucial for long-term usage. Designing a universal, sustainable, nontoxic, and biodegradable adsorbent with increased adsorption capacity can reduce the need for adsorbents, acid/alkali for regeneration, and overall recycling requirements. As these biocomposites become more resilient and functional, they may create a new market. Continuous research into their performance and life-cycle evaluation is necessary to determine their usefulness in replacing conventional harmful nonbiodegradable materials in the near future.

Nevertheless, sustainability concerns regarding renewable resources in composite materials necessitate concerted efforts from academia, industry, and government to develop economically feasible and environmentally sustainable alternatives. Advancements in extraction processes for manufacturing new biobased materials on an industrial scale are paramount to meet the increasing demands of society, especially in rural areas. Efforts should focus on improving processability, commercial scale structure, cost-effectiveness, efficiency, stability, and environmental impact to enhance competitiveness and replace existing technologies entirely.

Further advancements in regeneration techniques for cellulose and chitosan-based resins will contribute significantly to their sustainability. Efficient regeneration processes enable multiple cycles of use, thereby reducing operational costs and minimizing environmental impact. In conclusion, collaborative efforts involving industries, governments, nongovernmental organizations, and academia are essential to translate laboratory-scale research into industrial-scale solutions, ensuring access to economic clean water for human consumption on a global scale.
